# Machine Learning-Assisted SERS Reveals the Biochemical Signature of Enhanced Protein Secretion from Surface-Modified Magnetic Nanoparticles

**DOI:** 10.1021/acsami.4c18591

**Published:** 2024-12-11

**Authors:** Ibrahim Dagci, Kubra Solak, Nazli Oncer, Seyda Yildiz Arslan, Yagmur Unver, Mehmet Yilmaz, Ahmet Mavi

**Affiliations:** † Department of Molecular Biology and Genetics, Institute of Science and Technology, Atatürk University, 25240 Erzurum, Türkiye; ‡ East Anatolia High Technology Application and Research Center (DAYTAM), Atatürk University, 25240 Erzurum, Türkiye; § Department of Nanoscience and Nanoengineering, Institute of Science and Technology, Atatürk University, 25240 Erzurum, Türkiye; ∥ Department of Molecular Biology and Genetics, Faculty of Science, Atatürk University, 25240 Erzurum, Türkiye; ⊥ Department of Chemical Engineering, Atatürk University, 25240 Erzurum, Türkiye; # Department of Mathematics and Science Education, Education Faculty of Kazim Karabekir, Atatürk University, 25240 Erzurum, Türkiye

**Keywords:** iron oxide nanoparticles, magnetic immobilization, recombinant protein, Komagataella phaffii, SERS, machine learning

## Abstract

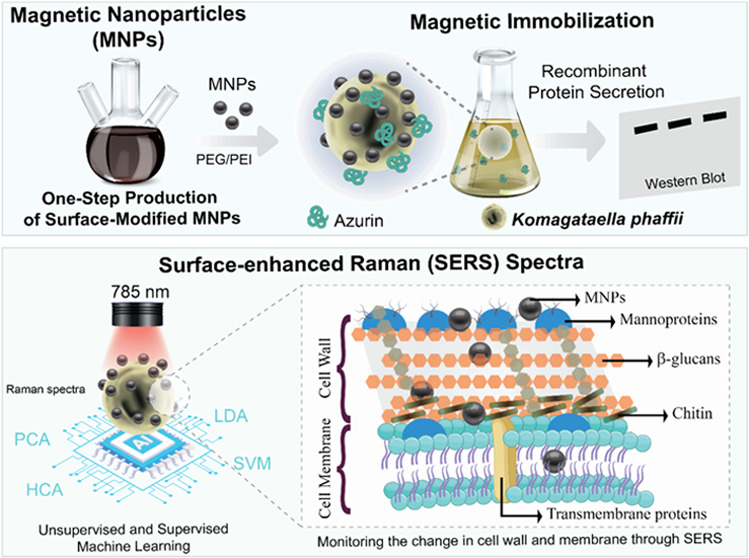

This study introduces a novel investigation of the interaction between *Komagataella phaffii* cells and iron oxide-based magnetic nanoparticles (Fe_3_O_4_ MNPs) via protein secretion and machine learning (ML)-assisted surface-enhanced Raman scattering (SERS). For the first time, we produced Fe_3_O_4_, Fe_3_O_4_@PEG, Fe_3_O_4_@PEI_10kDa_, and Fe_3_O_4_@PEI_25kDa_ MNPs by a one-pot coprecipitation reaction. The addition of polymers to the reaction conditions significantly affected the shape, surface charge, size, and size distribution of the MNPs. The surface modification of MNPs is effectively accomplished using polyethylenimine (PEI), and the ζ-potential values of the MNPs exceed +25 mV under the NH_4_OH control. The homogeneity of MNPs synthesized with NH_4_OH is more pronounced according to transmission electron microscopy (TEM) pictures. All MNPs exhibited excellent immobilization efficiency (>92%) when we used 250 ppm Fe-containing MNP solutions. Smaller MNPs uniformly encapsulated the surface of *K. phaffii* cells, whereas larger MNPs exhibited irregular accumulation. *K. phaffii* cells exhibited excellent viability in all MNP solutions at up to 1000 ppm of Fe concentrations. Finally, the highest recombinant azurin protein secretion rate was obtained in Fe_3_O_4_@PEI_10kDa_ MNP-immobilized cells (about 1.3 times). The ML-assisted SERS analysis revealed that MNP interactions with *K. phaffii* cells were mediated by proteins such as mannoproteins and membrane transporter proteins as well as N-acetylglucosamine (i.e., chitin). These findings revealed the effect of the size and surface properties of MNPs on the immobilization of *K. phaffii* cells and the enormous potential of magnetic immobilization for protein secretion.

## Introduction

Industrial-scale recombinant protein production has a substantial portion of the biotechnology market, and an increasing number of biopharmaceutical products are being approved.^1^ Small eukaryotic yeast cells, such as *Komagataella phaffii* (previously described as *Pichia pastoris*), are distinguished by their capacity to produce recombinant proteins due to their numerous advantages, including high quality, stable, and high levels of intracellular and extracellular protein expression, large-scale production at minimal cost, and the ability to perform higher levels of eukaryotic protein modifications (glycosylation, etc.).^2^*K. phaffii* has produced complex heterologous proteins or biostructures such as eukaryotic ATP-dependent transporters, human interleukin-3 (hIL-3), anticancer bacterial azurin protein, and HBcAg virus-like particles.^3−7^ The α-factor signaling sequence in the expression plasmid allows most proteins generated in the *K. phaffii* expression system to be released into the culture medium. However, a certain amount of the proteins produced remains inside the cell.^7,8^ Therefore, employing methods that enhance the extracellular release of the synthesized protein will provide a substantial benefit in terms of both the increase in the quantity of recombinant protein obtained from the culture medium and the elimination of the requirement for cell lysis during protein purification. For this, the immobilization methods are frequently being examined to facilitate the recovery of cells from the culture media and their subsequent reuse, as well as to optimize the release of extracellular proteins.^9^

Magnetic immobilization with magnetic nanoparticles (MNPs) provides many advantages, such as easy collection from suspension and reuse of cells with a simple magnet,^9^ high yields,^10^ and increased resistance of cells to environmental factors^11^ and activity.^12^ Magnetic immobilization also increases the release of intracellular proteins into the culture medium by affecting membrane permeability.^10^ The surface properties of MNPs play an important role in immobilizing yeast cells.^13−15^

MNPs are rapidly produced by coprecipitation reaction using different precursors or base sources to control the properties of MNPs such as surface, size, and shape.^16^ Studies comparing the biological activities of MNPs produced in one pot containing different base sources and polymers are limited. Investigating the impacts of the physical and chemical properties of MNPs on the cellular immobilization process is crucial since the immobilization process is strictly dependent on these properties. It also has an impact on the stability and efficiency of the immobilization process. Conventional methods such as flow cytometry, atomic force microscopy (AFM), scanning electron microscopy (SEM), fluorescent/confocal microscopy, and Fourier transform infrared spectroscopy (FTIR) are frequently employed to investigate the interaction of NPs with cells.^17^ Nevertheless, these technologies are inadequate at providing sufficient information regarding biomolecular signatures. As a direct molecular fingerprint method, the Raman spectrum accurately and promptly identifies the chemical composition of the bacteria, yeasts, and other cells.^18^ However, owing to its low Raman cross section (∼10^–30^ cm^2^.molecule^–1^), the Raman effect is feeble, creating limitations in the identification of biomolecules at extremely low levels and in distinguishing between the intricate overlapping patterns of biomolecules.^19−21^ In the past 40 years, the surface-enhanced Raman scattering (SERS) method has been developed and utilized to overcome these limitations.^20−22^ In order to enhance the repeatability and reliability of SERS signal amplification, a variety of SERS substrates have been developed, including nanoparticles (NPs), nanorods, nanowires, nanoshells, nanoholes, core–shell structures, and three-dimensional surfaces.^21,23−25^ As a powerful technique, the SERS may provide unique opportunities to reveal the interaction of yeast cells with NPs, with its high sensitivity, specificity, reliability, usefulness, and label-free detection ability.^26^ The primary focus of SERS investigations mainly focused on the identification of species.^26−28^ The comparison of SERS spectra from *Candida* species decorated with silver NPs facilitates species identification by detecting distinct components of the yeast cell wall.^26^ The presence of strong mannoproteins in the yeast cell wall was determined by analyzing a single live *Saccharomyces cerevisiae* cell using SERS spectral imaging.^29^ Another use of SERS is the separation of wild-type *S. cerevisiae* from yeasts that produce recombinant proteins based on their response to antifungal agents.^21^ However, complicated SERS spectra are analyzed using machine learning (ML) techniques, which reveal crucial properties that help identify and differentiate species or molecules.^30^

**Scheme 1:**
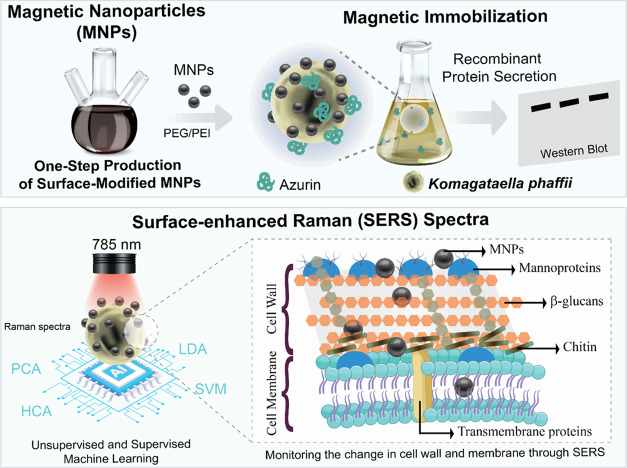
Summary of the Study Bare and modified MNPs were produced and characterized to evaluate their effectiveness in immobilizing the *K. phaffii*. The interaction of MNPs with the yeast (*K. phaffii*) surface was investigated by machine learning-assisted SERS. The recombinant azurin secretion from magnetically immobilized *K. phaffii* cells was compared using Western blot analysis.

In this study, for the first time, the molecular interaction of the *K. phaffii* cells and MNPs produced and functionalized in a single step was investigated in terms of recombinant protein secretion and ML-assisted SERS (see Scheme 1). Western blot analysis quantitatively revealed the secretion of azurin protein, known for its potent anticancer properties, from *K. phaffii* cells were magnetically immobilized with different MNPs. We used electron microscopy and ML-assisted SERS to illuminate MNPs and the yeast cells interactions. Our SERS platform showed significantly high Raman activity, and ML techniques undoubtedly allowed us to distinguish biomolecular signatures between different sample groups.

## Experimental Procedures

### Materials

Iron(II) chloride tetrahydrate (FeCl_2_·4H_2_O), iron(III) chloride hexahydrate (FeCl_3_·6H_2_O), poly(ethylene glycol) 3500 (PEG), polyethylenimine, (PEI, branched, 25 kDa), ammonia solution (25–30% w/w, NH_4_OH), HCl (36.5–38.0%), biotin, yeast extract peptone dextrose (YPD), d-sorbitol, and glutaraldehyde (50 wt % in H_2_O) were purchased from Sigma-Aldrich. Sodium hydroxide (NaOH), yeast extract, and potassium dehydrate phosphate (KH_2_PO_4_) were obtained from Merck. Bacteriological peptone (Neogen), yeast nitrogen base (YNB), glycerol (ChemCruz), casamino acid (USBiological), agar (Neogen), and polyethylenimine (PEI, branched, 10 kDa, Polyscience) were used without any prior treatments.

### Synthesis and Characterization of MNPs

Iron oxide magnetic nanoparticles were synthesized by the coprecipitation method using FeCl_3_·6H_2_O and FeCl_2_·4H_2_O under a nitrogen atmosphere. The production process in the presence of NaOH was performed in a three-necked flask containing a 0.63 M NaOH solution (29 mL). The iron salts ([Fe^3+^]:[Fe^2+^] = 2:1 M, 2.5 mL each in water) were added to the boiling solution. Then, 0.001 M of the coating molecule (PEG, PEI_10kDa_, PEI_25kDa_) dissolved in an aqueous solution (1 mL) or 1 mL of ultrapure water for bare MNPs was added to the mixture. In the production with NH_4_OH, iron salts were dissolved in 29 mL of ultrapure water and boiled before adding 5 mL of NH_4_OH solution (25–30%). Then, 1 mL of pure water or a coating molecule dissolved in pure water (PEG, PEI_10kDa_, PEI_25kDa_) was added. All reactions were boiled for 2 h and cooled to room temperature before magnetic separation. MNPs were washed several times with water and stored at +4 °C under a nitrogen atmosphere.

The MNPs were characterized by several techniques such as transmission electron microscopy (TEM, Hitachi, HighTech HT7700), X-ray diffraction (XRD, PANalytical Empyrean, Malvern), Fourier transform infrared spectroscopy (FTIR, Bruker VERTEX 70 v), and dynamic light scattering (DLS, Malvern Zetasizer Nano ZSP).

XRD analysis revealed the crystalline nature and phase composition of the MNPs. The analysis was performed using Cu Kα radiation (λ = 1.5406 Å) over a range of 10–90° with a step size of 0.02° under operating conditions of 40 mA and 45 kV. The crystal sizes were calculated using the Scherrer equation. The equation is formulated as follows:
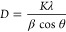
where *D* is the crystal size, *K* is the Scherrer constant (typically around 0.9), λ is the wavelength of the X-rays, β is the full width at half-maximum (fwhm) of the peak, and θ is the Bragg angle.

FTIR spectroscopy elucidated the functional groups and surface chemistry of the MNPs. Samples were scanned in ATR mode within the 400–4000 cm^–1^ range.

TEM imaging provided high-resolution insights into the morphology and size distribution of the MNPs. The samples were dropped onto a carbon-coated copper grid and air-dried under standard room conditions before examination at 120 kV.

Before the ζ-potential measurement of the MNPs, the MNPs were highly diluted with water, and then, the sample was charged into a scattering cell at 25 °C.

Inductively coupled plasma mass spectrometry (Agilent 7800 Quadrupole ICP-MS, Agilent Technologies) was used to quantify the iron content in samples. Before measurement, the samples were exposed to sequential acid and microwave digestion.

### Cultivation and Magnetic Immobilization of Cells

The recombinant *K. phaffii* cells expressing the azurin protein were available in our laboratory.^7^ YPD agar was prepared by dissolving 5 g of YPD and 2 g of agar in 100 mL of purified water and sterilized by autoclaving at 121 °C for 15 min. Preculture (BMGY, Buffered Glycerol Complex Medium) and production (BMMY, Buffered Methanol Complex Medium) media were prepared in the baffled flask. BMGY medium was obtained, containing 1% yeast extract, 2% peptone, 1% KH_2_PO_4_ (100 mM), 1.34% YNB, 1% glycerol, and 25 μL of 500X B (0.02% Biotin). BMMY medium was prepared with the following ingredients: 1% yeast extract, 2% peptone, 1% KH_2_PO_4_ (100 mM), 1.34% YNB, 1% sorbitol, 50 μL of 500X B, and 1% casamino acid. In both media, peptone, and yeast extract were first autoclaved, then other components were added to the media after filtering (0.22 μm).

Recombinant *K. phaffii* cells streaked on YPD agar were incubated at 30 °C for 48–60 h. A loop of yeast cells was inoculated into 12.5 mL of BMGY medium and incubated at 30 °C and 225 rpm overnight. Distilled water was used twice to wash the cells, following centrifugation. The cell pellets dispersed in 5 mL of medium were inoculated into 20 mL of BMMY medium, and the culture was incubated at 30 °C and 280 rpm. For promoter induction and azurin production, methanol was added to the production medium every 24 h to a final concentration of 0.5% (v/v).

*K. phaffii* cells were grown in BMGY medium until they reached an optical density (OD) of 4 at 600 nm (OD_600_, ultraviolet (UV)-spectrophotometer, Shimadzu, Kyoto, Japan). For each immobilization, 5 mL of culture was centrifuged at 4000 rpm for 5 min. The cells were washed with 5 mL of a 0.9% physiological saline and resuspended in 5 mL of this solution.^9,10,15^ For magnetic immobilization, 5 mL of MNP solution with a final Fe content of 250 ppm was added to yeast cells and incubated at 30 °C and 150 rpm for 15 min. Immobilized cells were collected with a magnet. The supernatant was used for OD_600_ measurement to determine immobilization efficiency. After the cell pellets were suspended in 25 mL of BMMY medium, the culture was incubated at 30 °C and 280 rpm for 72 h. Finally, 0.5% (v/v) methanol was added to the culture every 24 h for the promoter induction of azurin expression. For cytotoxicity assay, magnetically immobilized *K. phaffii* cells were grown in YPD medium for 72 h, and the OD_600_ (BioTek Epoch Microplate Spectrophotometer) was measured by taking samples every 24 h.

### Interaction of Nanoparticles with Cells

Scanning electron microscopy (SEM) demonstrated how *K. phaffii* cells were coated with MNPs. After immobilization, the cells were smeared onto a glass slide (cleaned by sonication in 1% Triton X-100, distilled water, and 70% ethanol for 20 min in succession) and allowed to air-dry at room temperature. They were fixed in 2 mL of 2.5% glutaraldehyde for 45 min.^10^ After rinsing with 0.9% physiological saline (15 min), the slides were dehydrated by incubating them in 30, 50, 70, 80, and 90% ethanol for 10 min, respectively. The slides were incubated in 100% ethanol for 20 min to accomplish the last dehydration step. The ethanol was entirely removed from the slides by incubating them at room temperature overnight. Samples were sputter-coated (Quorum, Q150R S) with gold for 150 s at 20 mA. Then, SEM (Zeiss, Sigma 300) images were collected under high vacuum at 5000× magnification.

### Western Blot Analysis

The supernatants were collected from immobilized yeasts harboring (i) and not harboring (ii) the azurin gene and (iii) nonimmobilized yeasts harboring the azurin gene. Sodium dodecyl-sulfate polyacrylamide gel electrophoresis (SDS-PAGE) (Mini-Protean, Bio-Rad) was performed using the culture supernatants combined with an equivalent volume of 1× SDS loading buffer.^31^ Following electrophoresis, proteins were transferred from the gel to a poly(vinylidene difluoride) (PVDF) membrane. The membrane was blocked with phosphate-buffered saline with Tween 20 (PBST) containing 5% skim milk powder, then incubated with antibodies (antiazurin and antigoat IgG H&L (HRP), 1:6000) as described in the previous study.^7^ Protein bands were visualized using Supersignal West Femto and Pico chemiluminescent substrates (Pierce Biotechnology, Rockford, IL, Thermo Scientific) and imaged using the ChemiDoc Touch Imaging System (Bio-Rad).

### SERS Analysis

In this study, we employed gold nanorod arrays (GNAs) as a SERS platform, which were fabricated via the physical vapor deposition (PVD) method and analyzed in previous studies in detail.^20,32−34^ Magnetically immobilized cells (5 μL, OD_600_: 0.5) were air-dried on the GNAs substrate. Raman spectra (2000–200 cm^–1^) were obtained using a WITEC α 300R Micro Raman microscope using a 785 nm laser source. The laser power and the application time were set to 10 mW and 5 s, respectively. Many spectra with a high signal-to-noise ratio for each sample were collected, and randomly selected spectra were further analyzed using machine learning (ML) methods.

Baseline correction (Rubber band correction with 64 baseline points), smoothing (with 13 smoothing points), and normalization (min-max normalization) were performed as spectral preprocessing on the raw spectra by using OPUS 5.5 (OPUS, Bruker Optics) software. Multivariate ML analyses were then applied using Unscrambler X 10.4. Principal component analysis (PCA) and hierarchical cluster analysis (HCA) were used as unsupervised ML analyses. The results were presented as score and loading plots using the full cross-validation method in PCA and as a dendrogram plot like a family tree in HCA. The supervised ML techniques of linear discriminant analysis (LDA) and the support vector machine (SVM) were employed to test the reliability of the classification model and the performance of GNA as a SERS platform. Before these analyses were conducted, two spectra were randomly chosen from each group and designated as blind test samples. In LDA, a PCA training set and a random test set were generated for each sample, and the analysis was performed accordingly. In SVM, a PCA training model was created for each sample, and analysis was performed. LDA and SVM analyses are presented as scatter plots. Randomly selected test sets were used to perform LDA and SVM classifications, and the results were presented in tables. Each test was repeated five times, and average accuracy scores were obtained for supervised analysis.

### Statistical Analysis

OriginPro version 10.1–2024 was used for all statistical analysis and data visualization. Western blot band quantitative analysis was performed using ImageJ software. A statistically significant difference was defined as n.s. (not significant, *p* ≥ 0.05), * *p* < 0.05, ** *p* < 0.01, and *** *p* < 0.001 between the groups.

## Results and Discussion

### One-Pot Coprecipitation Reactions Are a Simple Way to Create Surface-Modified Nanoparticles

Iron oxide (Fe_3_O_4_) MNPs are usually synthesized with NH_4_OH or NaOH as the base source. NaOH accelerates the reaction and forms large MNPs since it has a high concentration of OH̅ and a high pH value (pH 13). In contrast, NH_4_OH helps to control particle size by relatively lower OH̅ releasing. Adding NH_4_OH offers excellent stability and control compared to NaOH, facilitating a more precise adjustment of pH levels.^35^ All of these variables influence the crystalline and magnetic characteristics of the generated MNPs. Thus, the synthesis conditions significantly impact how these MNPs interact with biological systems. In various studies, bare Fe_3_O_4_ MNP is first produced, and subsequently, the surface of MNPs is modified by using polymers.^12,36^ Modifying the surface of the MNPs with surface-modifying agents at the same time of production enables control of the shape, size, and surface properties of the MNP in a single step. In this work, bare, PEI-, and PEG-modified MNPs were synthesized in a single-step coprecipitation method and characterized in detail.

**Figure 1:**
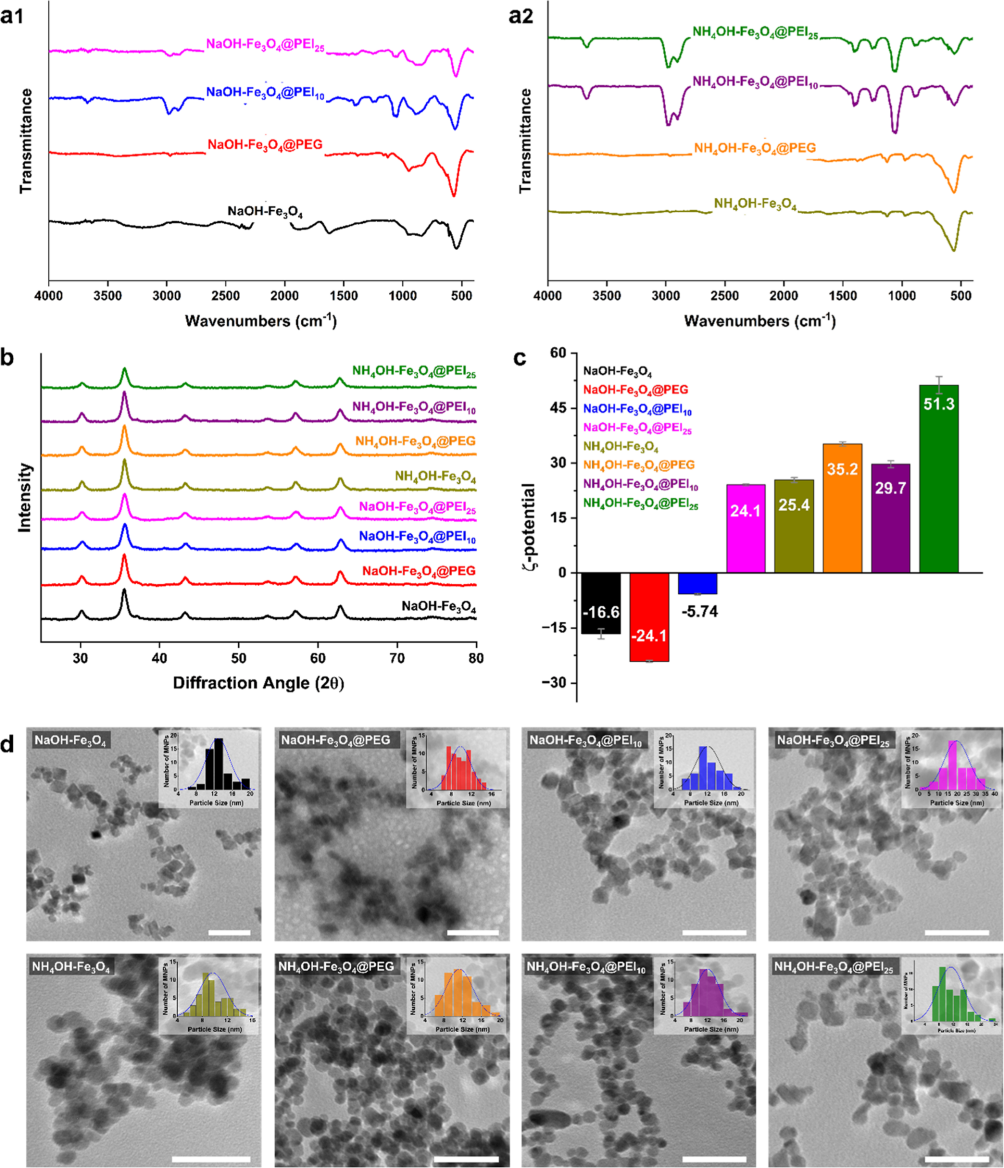
Characterization of surface-modified MNPs. (a1, a2) FTIR spectra, (b) XRD pattern, (c) ζ-potential, and (d) TEM images of surface-modified MNPs. The scale bar is 50 nm.

First, FTIR spectroscopy was used to determine the chemical bonds and functional groups in the MNPs. The peak originating from the stretching of Fe–O bonds was observed at ∼560 cm^–1^ in all samples (see Figure 1a).^37^ When the bare Fe_3_O_4_ MNPs were compared, the peak of −OH groups was observed prominently at around 3200 cm^–1^ in the NaOH–Fe_3_O_4_ than NH_4_OH–Fe_3_O_4_ in agreement with previous studies.^37^ This peak indicates that NaOH enables the formation of −OH groups on the surface of the MNPs during the reaction. The peak at 1622 cm^–1^, which is thought to belong to adsorbed water, was observed in both MNPs. The peak at 1622 cm^–1^ observed in NaOH–Fe_3_O_4_ MNPs was absent in the NaOH-Fe_3_O_4_@PEG MNPs. The peaks at 1130 and 1161 cm^–1^ appeared in NaOH-Fe_3_O_4_@PEG MNPs, which were attributed to C–O stretch.^37^ The peaks at 3674, 2889, 2973, 1393, 1393, 1251, 1234, 1043, and 879 cm^–1^ indicate that the PEI polymer is immobilized onto the MNP surface in both NaOH and NH_4_OH containing reaction conditions to obtain Fe_3_O_4_@PEI_10_ and Fe_3_O_4_@PEI_25_ MNPs. In the spectra, it was noticed that peaks that indicated the presence of PEI were more pronounced in the case of the NH_4_OH-based synthesis. This may be because NH_4_OH allows the branched PEI in solution to reach a higher aspect ratio.^38^ The double peaks around ∼2880, and ∼2990 cm^–1^ were attributed to the C–H structure and were detected prominently in the samples containing PEI, while these peaks were slightly observed in the MNPs produced with PEG. The FTIR data, which shows peaks associated with Fe–O bonding and surface modifications in MNPs, validate the generation of surface-modified MNPs in line with previous research.^37,39^

The prominent peaks noticed in the XRD spectra of the MNPs were located at specific 2θ angles of approximately 30.1, 35.5, 43.2, 53.6, and 57.1° (Figure 1b). These diffraction angles were associated with the crystallographic planes indexed as (220), (311), (400), (422), (511), and (440), respectively, conforming to the structural arrangement of magnetite. The α-Fe_2_O_3_ phase observed in XRD data reduces when Fe_3_O_4_ MNPs are coated with a polymer such as PEG, according to previously published studies.^37^ The diffraction patterns of all MNPs are almost identical, indicating that the crystal phase of the Fe_3_O_4_ MNPs did not change when polymers were added to the reaction conditions. This demonstrates that MNP production forms a single crystal form consistent with magnetite’s structural arrangement, and different oxidation states do not occur. According to Scherrer’s Equation, calculated using the fwhm values obtained from the peak at 35.5°, the average crystallite sizes for MNPs produced using NaOH and NH_4_OH were found to be 9.96, 9.69, 8.42, 9.02, 10.30, 10.02, 10.37, and 10.14 nm for NaOH–Fe_3_O_4_, NaOH–Fe_3_O_4_@PEG, NaOH–Fe_3_O_4_@PEI_10_, NaOH–Fe_3_O_4_@PEI_25_, NH_4_OH–Fe_3_O_4_, NH_4_OH–Fe_3_O_4_@PEG, NH_4_OH–Fe_3_O_4_@PEI_10_, and NH_4_OH–Fe_3_O_4_@PEI_25_, respectively. The production of MNPs with NH_4_OH yields an average crystal size of approximately 10 nm, whereas NaOH yields an average crystal size of approximately 9 nm.

Dynamic light scattering (DLS) measurement was used to determine the size distribution of the MNPs (PDI) in solution, the hydrodynamic radius (*R*_h_), and the surface charges (ζ-potential) of the MNPs. All MNPs produced in the presence of NH_4_OH have ζ-potential values > +25 mV (see Figure 1c). Positive ζ-potential was determined when only 25 kDa of PEI was used in production with NaOH. This is a highly expected result for MNPs whose surface is modified with polymers. Based on our synthesis conditions, PEI is a more appropriate polymer for surface modification of MNPs than PEG. It was reported that the concentration of PEG_4000_ had a significant effect on MNP size and magnetic properties.^35^ The FTIR spectrum of NaOH–Fe_3_O_4_@PEI_10_ MNPs indicates the presence of PEI in the structure, but surprisingly, the observed ζ-potential value is negative. This demonstrates that the base and polymer directly influence the deposition of the polymer on the surface of the MNPs. This situation will significantly affect the use of MNPs in biological applications. Also, according to *R*_h_ and PDI values (see Figure S1), the most unstable MNPs with the highest aggregation tendency were produced using PEI in the presence of NaOH. It was determined that the samples with the lowest *R*_h_ size were NaOH–Fe_3_O_4_@PEG, NaOH–Fe_3_O_4_, and NH_4_OH–Fe_3_O_4_@PEI_25_ MNPs with 112.2 ± 0.57, 142.7 ± 1.301, and 176.9 ± 2.381 nm, respectively. These MNPs are also observed to have the lowest PDI. Thus, it is predicted to facilitate the interaction of produced MNPs with the surface structures of cells such as yeast. The MNPs were dispersed in distilled water after sonication, and the resulting suspension responded to an external magnetic field, as shown in Figure S2.

Finally, TEM images of MNPs show that using NH_4_OH in MNP synthesis leads to the production of MNPs with a more uniform shape (see Figure 1d). The information in the literature that NaOH causes a higher size of MNPs than NH_4_OH is supported by our findings.^40^ It was observed that MNPs had a pronounced tendency to aggregate when high-molecular-weight PEI was used. The morphology and size of MNPs were found to be easily manipulated by altering the base source when PEG was used as a polymer. It was determined that MNPs in the solution containing PEG and NH_4_OH had a regular morphology and homogeneous size distribution. High-molecular-weight PEI caused the spherical MNP morphology to shift to an oval shape in the presence of NH_4_OH. In general, a decrease in the size of the MNP was observed upon adding the polymer to the reaction medium or increasing the polymer length. The growth of the MNP may be restricted due to the polymer’s inhibition of the new Fe–O interaction during synthesis.^37^ In addition, using surfactants for size control reduces particle sizes by restricting oxidation.^41^ The sizes of NaOH–Fe_3_O_4_, NaOH–Fe_3_O_4_@PEG, NaOH–Fe_3_O_4_@PEI_10_, NaOH–Fe_3_O_4_@PEI_25_, NH_4_OH–Fe_3_O_4_, NH_4_OH–Fe_3_O_4_@PEG, NH_4_OH–Fe_3_O_4_@PEI_10_, and NH_4_OH–Fe_3_O_4_@PEI_25_ MNPs were 12.8 ± 2.6, 9.8 ± 2.1, 12.2 ± 2.9, 19.7 ± 6.6, 9.7 ± 2.1, 11.3 ± 2.8, 12.1 ± 2.9, and 11.4 ± 3.7 nm, respectively.

In conclusion, magnetic Fe_3_O_4_ MNPs were successfully synthesized. The incorporation of polymers into the synthesis environment allows for the deposition of these polymers onto the MNP surfaces while, at the same time, altering the characteristics of the MNPs, such as their size, shape, and dispersion in water.

### Surface-Modified Magnetic Nanoparticles Are Able to Decorate the Surface of *K. phaffii* Cells

Surface-modified MNPs were used to immobilize the *K. phaffii* cells, which were collected using a magnet. Immobilization efficiency was determined by measuring the supernatant’s optical density at 600 nm (OD_600_). The new ability to magnetize *K. phaffii* cells and separate them from the media containing the recombinant product by a magnet is a pioneering method that will accelerate the processes of purifying recombinant proteins. Indeed, yeasts are eukaryotic microbes with a single cell that is essential in the fermentation process.^7,17^ The manufacture of bioethanol, medicines, and dietary supplements in bioreactors is among the many industrial uses of yeasts.^2^ The massive production of heterologous proteins by *K. phaffii* cells makes it an important model organism.^8,42^ Here, excellent immobilization effectiveness (>92%) was noted when all samples were immobilized with MNPs at a concentration of 250 ppm Fe. In the literature, PEI-modified MNPs have been used for the isolation of microorganisms and exhibit high immobilization efficiency.^12,36^ Using amine-modified MNPs, the magnetic immobilization was performed of *K. phaffii* was reported to be highly effective.^9,10,15^

**Figure 2:**
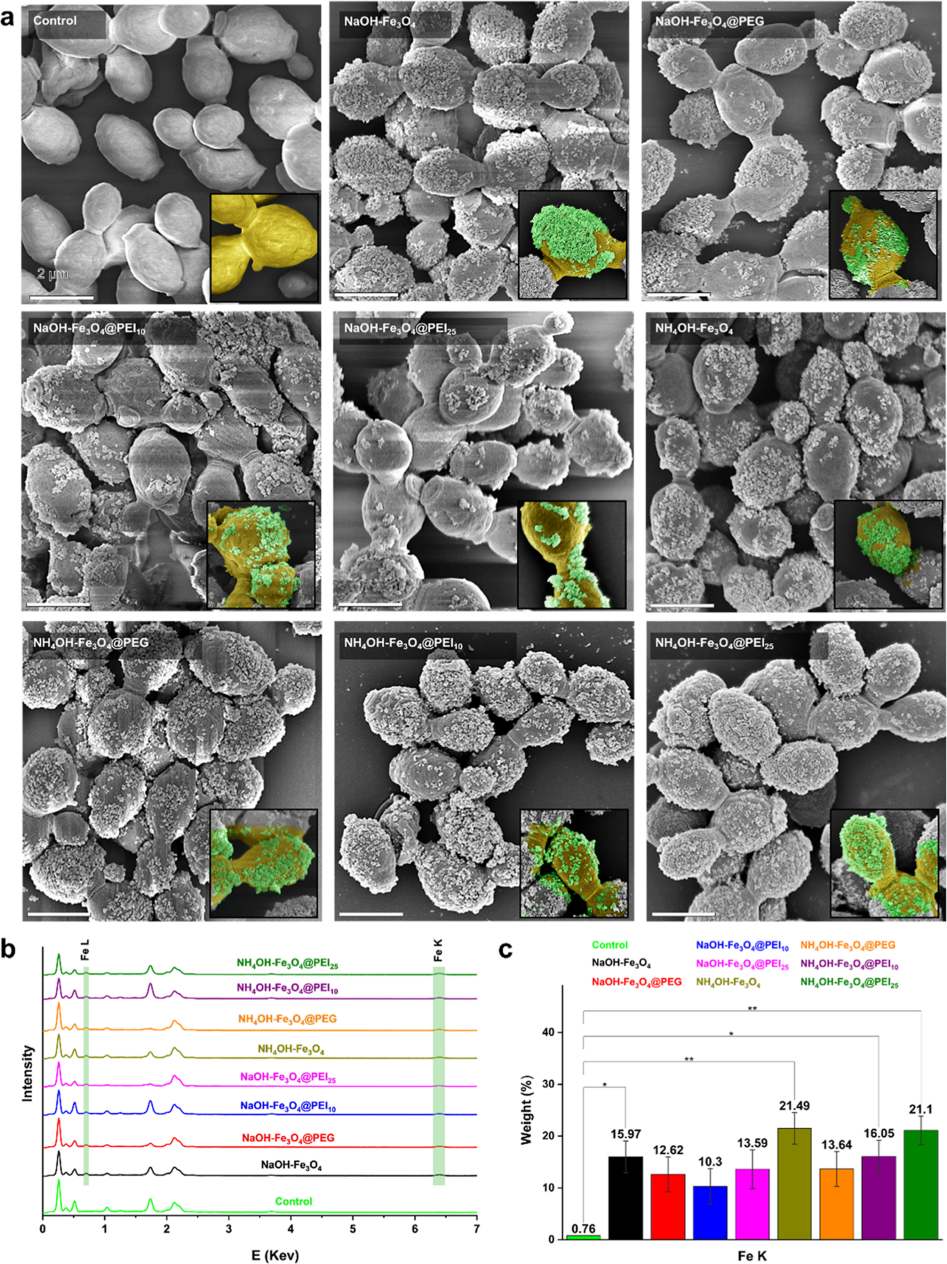
Interaction of surface-modified MNPs with the surface of *K. phaffii* cells: (a) SEM and pseudocolor SEM images, (b) EDX spectrum and (c) weight percent ratios of iron (Fe K) on the surface of magnetically immobilized cells. *K. phaffii* was labeled in yellow pseudocolor and MNPs in green pseudocolor. Immobilization was accomplished by incubating cells in a medium containing 250 ppm Fe for 15 min. The scale bar is 2 μm.* *p* < 0.05, ** *p* < 0.01, *** *p* < 0.001.

In this study, the surface of *K. phaffii* cells was successfully decorated with all types of MNPs, as demonstrated by the pseudocoloring of SEM images (see Figure 2a). Immobilization with MNPs without any polymer modification (NaOH–Fe_3_O_4_ and NH_4_OH–Fe_3_O_4_) showed that yeast surfaces were covered entirely with MNPs like a shell. However, there is more localized immobilization in the PEI-modified MNPs. EDX analysis revealed an increase in the amount of Fe on the immobilized cell surfaces compared to the control, which are nonimmobilized *K. phaffii* cells (see Figure 2b,2c). The Fe ratios determined according to the EDX measurements were correlated with the corresponding SEM images. The highest Fe content was recorded in Fe_3_O_4_ MNPs in the production of both base sources. This study determined that less Fe accumulated on the cell surface immobilized with polymer-modified MNPs than on naked MNPs. This indicates that the modified MNPs have less interaction with yeast cells. The difference may be attributed to the length or functional groups of the polymers. Naked MNPs have a reduced *R*_h_ (Figure S1) and increased cellular interaction due to the absence of steric restriction. The small interaction between yeast and MNPs with a large *R*_h_ (2452 ± 213.6 and 4803 ± 511.4 nm for NaOH–Fe_3_O_4_@PEI_10_ and NaOH–Fe_3_O_4_@PEI_25_ MNPs, respectively) is probably due to the polymers causing aggregation and making it difficult to interact with cells. On the contrary, the immobilized cells with the higher Fe content were determined when NH_4_OH–Fe_3_O_4_ MNPs were used for immobilization. Surface energy-driven aggregation might cause a significantly higher *R*_h_ (709.2 ± 15.2 nm) on these NaOH–Fe_3_O_4_ MNPs.^43^ In this case, NaOH–Fe_3_O_4_ MNPs may interact with yeast surface proteins in a liquid medium containing yeast and tend to separate from each other. However, size and the pH-dependent electrostatic interactions also significantly influence how MNPs agglomerate.^44^ In the case of polymer modification, the polymer may prevent this interaction and dispersion of MNPs. So, in cells attached to polymer-modified MNPs, MNPs gathered in groups, while unmodified MNPs formed a shell around the yeast surface.

The surface morphology and structural properties of MNPs can affect the ability of cells to adhere and grow.^12^ These modifications improve interactions with targeted cells, support the immobilization of cells, and increase biocompatibility. The surface charge of MNPs influences electrostatic interactions with target cells. Positively or negatively charged surfaces have a dramatic role in the immobilization of cells, affecting the stability and efficiency of immobilized cells.^12^ Yeast surfaces immobilized with MNPs produced by NH_4_OH often showed more significant Fe contents (see Figure 2b,2c). This might be attributed to the correlation between the negatively charged surface of yeast and the significantly positive charge of MNPs generated in the presence of NH_4_OH.^45^ The resultant differences in electrical charge may positively impact the interaction. However, the groups with statistically significant Fe content changes on *K. phaffii* cell surface compared to control are cells immobilized with NaOH–Fe_3_O_4_ MNPs, NH_4_OH–Fe_3_O_4_ MNPs, NH_4_OH–Fe_3_O_4_@PEI_10_ MNPs, and NH_4_OH–Fe_3_O_4_@PEI_25_ MNPs. The presence of PEG or PEI did not significantly alter the quantity of Fe on the surface of *K. phaffii* cells in terms of immobilization with MNPs, which were synthesized using NaOH or NH_4_OH. This finding indicates that the quantity of MNP-immobilized on the yeast surface is not affected by the nature of the polymer. In addition to Fe, it was determined that the percentage of oxygen (O) also changed in the EDX analysis results (data not shown). These results indicate that the surface chemistry of the cells modified with iron oxide MNPs has changed significantly. Consequently, there is a direct correlation between *R*_h_ and the amount of Fe measured on the yeast surface. MNP synthesis conditions control the surface properties, size, and aggregation of MNPs, all of which have a significant influence on the MNP–yeast interaction. These changes may contribute to understanding material-cell interactions, but further analysis is needed to understand the biological interactions of the modified MNPs. Therefore, in this study, ML-assisted SERS analyses were employed to investigate the MNP–yeast surface interaction further.

Measurements of OD_600_ showed that *K. phaffii* cells remained viable in all MNP treatments, even at high dosages (see Figure S3). Although immobilization was performed with equal amounts of yeast cells, due to the black color of MNPs, a higher absorbance value was measured in media containing MNPs. An OD_600_ increase was observed at all incubation times and in all MNP treatments and maintained at all concentrations. It was determined that NH_4_OH–Fe_3_O_4_@PEG MNPs treatment was the only group not statistically different from the nonimmobilized control group. When the effect of incubation periods was examined, it was observed that there was a significant difference between the groups at 0–72 h. NH_4_OH–Fe_3_O_4_@PEI_10_ MNPs treatment was separated from all other groups at all application doses and times. The literature has demonstrated that growth inhibition, impairment of mitochondrial functions, and ROS-independent toxicity by targeting the respiratory chain complex IV occur in yeast cells treated with MNP at concentrations much higher than the MNP concentration (IC_50_ of the MNPs to yeast cell growth was 1353 ± 78 mg/L) used in this study.^46^ Future research may clarify the intracellular impacts of bare, PEG-, and PEI-modified MNPs.

### Magnetic Immobilization Increased Recombinant Azurin Secretion

**Figure 3:**
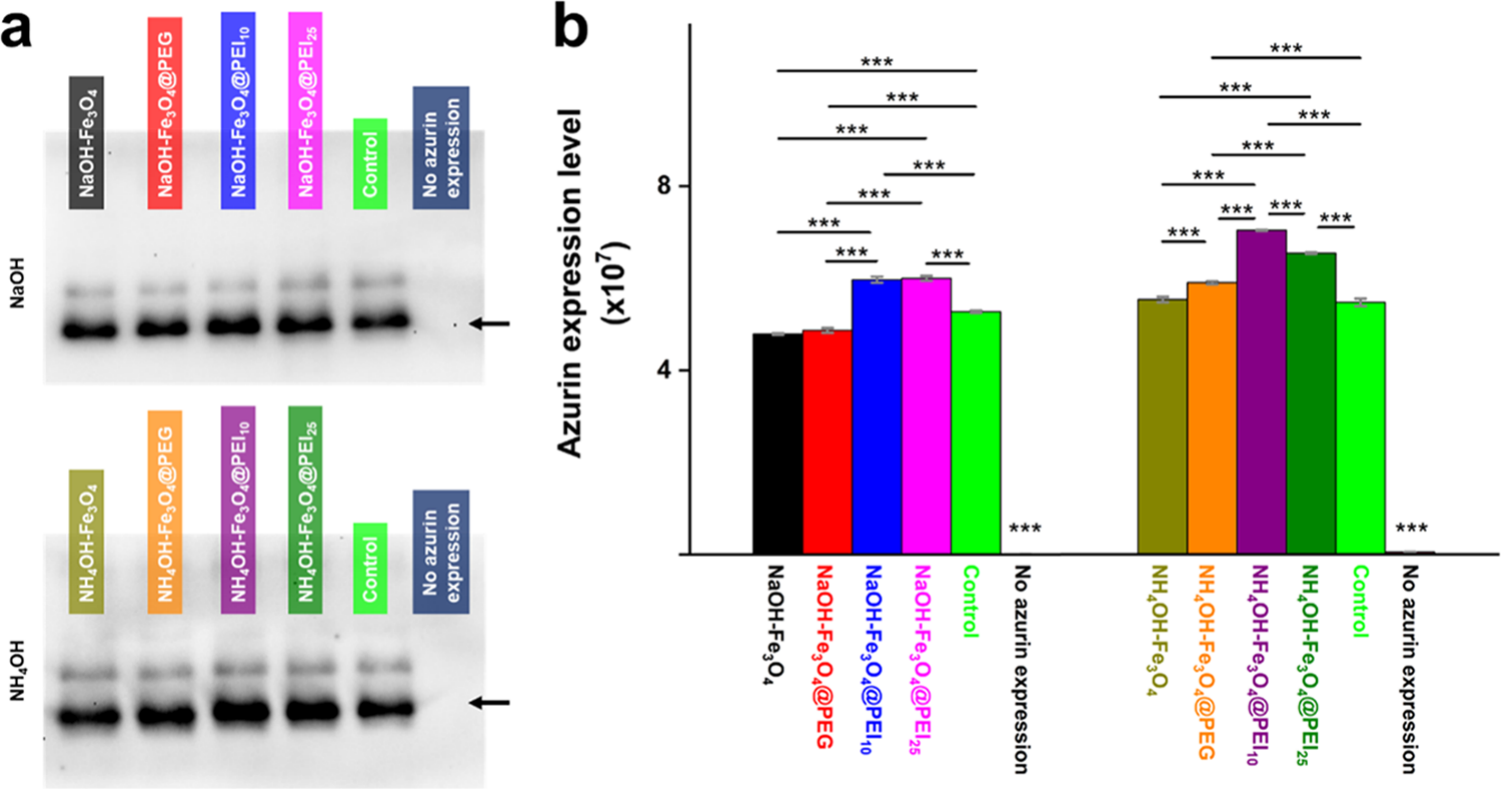
Expression analysis of azurin by magnetically immobilized *K. phaffii*. (a) Western blot membrane image. (b) ImageJ-calculated azurin band thicknesses. The arrow indicates the azurin. Control: Nonimmobilized cells. * *p* < 0.05, ** *p* < 0.01, *** *p* < 0.001.

The recombinant *K. phaffii* cells produce azurin in high yields under the control of the alcohol oxidase promoter.^7^ Additionally, it is known that magnetically immobilized *K. phaffii* cells increase extracellular secretion of target proteins.^9,10^ It was found that the amount of total protein in the production medium increased with time when it was calculated by sampling every 24 h for 72 h (data not shown). The results of Western blot analysis in Figure 3 show that PEI-modified MNPs increased azurin secretion at a level that was *** *p* < 0.001 compared to the nonimmobilized control cells. No significant difference was observed between the control and NH_4_OH–Fe_3_O_4_ MNPs. While there is no significant difference between the azurin bands of PEI-modified MNPs produced in the presence of NaOH, there is a remarkable difference between those produced with NH_4_OH (*** *p* < 0.001). The highest protein secretion was observed in NH_4_OH–Fe_3_O_4_@PEI_10_ MNP-immobilized at *K. phaffii* cells (1.34 ± 0.005-fold) compared to nonimmobilized control cells. When these data are combined with SEM images (see Figure 2), it can be concluded that the presence of MNPs on the surface of yeast cells, like a shell, may have a detrimental impact on protein release. An immobilization that covers the entire yeast surface, such as immobilization using Fe_3_O_4_ MNPs, may provide advantages in effectively collecting all yeasts from the culture medium. However, we demonstrated that a more localized immobilization, like that of Fe_3_O_4_@PEI MNPs, yields a higher benefit, in terms of protein secretion. According to our previous study, the recombinant *K. phaffii* cells had the highest secretion of the azurin to the production medium via the α-mating factor secretion signal.^7^ In this study, the secretion of recombinant azurin protein is examined in *K. phaffii* cells are enhanced by immobilization with MNPs, and the size and surface properties of MNPs are dominant factors in immobilization and protein secretion.

### Fabrication of SERS Substrate and Collection of Spectral Data

Before collecting SERS spectral data, we examined the morphology of the GNA system as a SERS platform through a detailed SEM analysis. As shown in Figure S4a, a three-dimensional (3D) and densely packed columnar anisotropic structure of GNAs was produced due to the angled deposition of gold, which could produce high SERS activity and enhancement factor.^20,21,32,34,47^ GNAs in 3D and anisotropic structures significantly increased the electric field. This is due to several phenomena, such as tip-focusing, nanoantenna effect, and light trapping, as proved in our previous reports.^21,48^ In particular, the use of GNA demonstrated significant SERS enhancement due to electromagnetic coupling effects and the formation of hot spots, as detailed in our earlier works.^20^ The employment of methylene blue as a Raman reporter molecule created a relative standard deviation (RSD) value lower than 0.17, demonstrating the acceptable reproducibility all over the GNA platform.^34^ The optical characteristics of the GNA SERS platform were assessed by using UV–visible (UV–vis) absorption spectra. The UV–vis absorption spectra of GNAs (Figure S4b) exhibited a large absorption peak spanning 400 to 600 nm, signifying the plasmonic characteristics of the GNA platform. Sensitive, reproducible, and high signal-intensity SERS spectra were collected from our GNA platform for various samples in our previous studies.^20,21,47,48^ This remarkable efficiency is due to the high SERS activity of GNAs, which serve as an ideal SERS system.^20,21^

**Figure 4:**
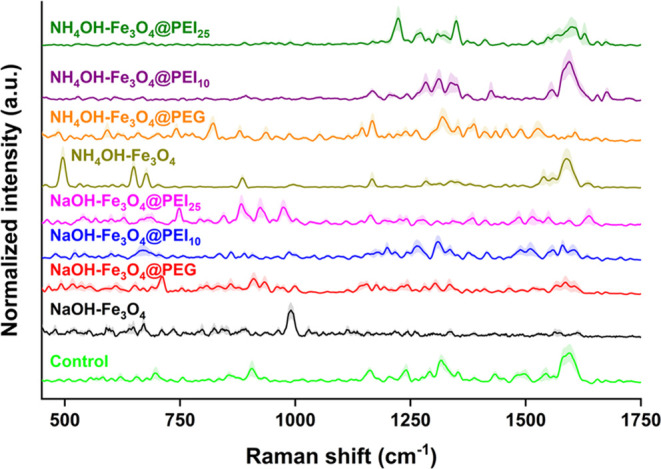
SERS results of magnetically immobilized *K. phaffii*. The average and standard deviation of SERS spectra of magnetically immobilized and nonimmobilized *K. phaffii* (5 s acquisition time, 10 mW laser power, 785 nm laser wavelength).

Collecting reliable and reproducible SERS spectral data with high signal intensity is essential to effectively obtain discrimination between different biological samples, including cells, bacteria, and yeasts. In one of our prior research, GNAs were used to identify recombinant yeast (*S. cerevisiae*) cells by SERS that had been transfected with the genes encoding the antiapoptotic proteins Bcl-2 and Mcl-1.^21^ The 3D anisotropic structure of the GNAs enhanced the electric field significantly. The GNA SERS platform exhibited excellent accuracy, sensitivity, and specificity in classifying yeast cells. Unsupervised machine learning techniques have been used to determine similarities and differences among recombinant fungal cells exposed to ketoconazole (KET) and fluconazole (FLU). Supervised machine learning techniques were utilized to determine and confirm the classification of fungal cells. We have successfully demonstrated that the antiapoptotic proteins Bcl-2 and Mcl-1 significantly enhanced cell survival compared to the empty vector group upon short-term treatment with KET and FLU. Consistent with our earlier research,^20,32,34^ we obtained robust SERS spectra from each yeast sample onto GNAs (see Figure 4). First, magnetically immobilized yeast cells were dropped onto GNAs to obtain SERS signals. The means and standard deviations of collected spectra from MNP-immobilized yeast samples are presented in Figure 4.^20,21^ Interestingly, we observed a clear difference even with the naked eye in the SERS peaks (average) of nonimmobilized yeast compared to magnetic-immobilized groups. The SERS peaks were obtained from *K. phaffii* cell surfaces modified with MNPs, such as NaOH–Fe_3_O_4_ and NH_4_OH–Fe_3_O_4_, which exhibited significant differences from the control. In instances of local MNP accumulation observed with NH_4_OH–Fe_3_O_4_@PEI_10_ and NH_4_OH–Fe_3_O_4_@PEI_25_ MNPs, SERS peaks comparable to the control were detectable on the *K. phaffii* cell surface. The SERS spectra of nonimmobilized *K. phaffii* exhibited strong peak intensities at 582, 694, 755, 855, 906, 1162, 1241, 1291, 1316, 1353, 1432, 1496, 1542, and 1595 cm^–1^. The peaks at 755 and 1432 cm^–1^ were assigned to vibrations of glycosidic ring and β-1,4N-acetylglucosamine, respectively.^26^ The 855 and 906 cm^–1^ peaks were attributed to β-glucan in the yeast wall.^49^ Also, glycosidic linkages were detected in the Raman spectra between 900 and 800 cm^–1^.^50^ The peaks at 582, 694, and 1162 cm^–1^ were assigned to the presence of tryptophan/cytosine and guanine, nucleic acids, and proteins, respectively.^51^ The Raman peaks in the control group at 1353, 1595, and 1542 cm^–1^ show that GNAs interact with mannoproteins of yeast surface in that region through oxygen-containing groups and nitrogen atoms.^29^ Three different Aspergillus species captured with MNPs exhibited significant Raman peak enhancements at 1218, 1326, 1457, and 1628 cm^–1^. Yeasts immobilized by NaOH–Fe_3_O_4_, NaOH–Fe_3_O_4_@PEI_10_, NaOH–Fe_3_O_4_@PEI_25_, and NH_4_OH–Fe_3_O_4_@PEG MNPs exhibit no signal in these regions, suggesting that mannoproteins facilitate the interaction between MNPs and *K. phaffii* cells, and it blocks the interaction of mannoproteins with GNAs. Research revealed that the SERS signal of Fe_3_O_4_@PEI.NH_2_ was significantly lower, and it attracts *Aspergillus* via electrostatic charge interactions.^52^ In conclusion, it has been suggested that MNPs made using NaOH (rather than NH_4_OH) prefer mannoproteins to interact with *K. phaffii* cells.

### Machine Learning-Assisted SERS Evaluation of MNP–*K. phaffii* Interaction

SERS is a powerful technique for detecting cells, biomolecules, and microorganisms due to its real-time, noninvasive imaging, low cost, and ability to acquire large amounts of data rapidly.^27,53^ Similar to the control group, we could obtain powerful SERS signals with a satisfying signal-to-noise ratio for the MNP-immobilized *K. phaffii* cells (see Figure 4). We could identify the variances for these samples, indicating robust SERS activity on the GNA platform. It seems that the immobilization of *K. phaffii* cells with MNPs created significant changes in the biomolecular content of cells and the resultant SERS spectra. However, despite the high variance in the spectral data, interpreting it becomes more difficult as the quantity and magnitude of data increase. Therefore, conducting these analyses by a researcher necessitates substantial effort and considerable time.^53,54^ To deal with these complexities, ML methods are frequently used to analyze and extract reliable information from complex spectral data. In the following sections, we employed various unsupervised and supervised ML techniques to discriminate the spectral data and test their reliability.

**Figure 5:**
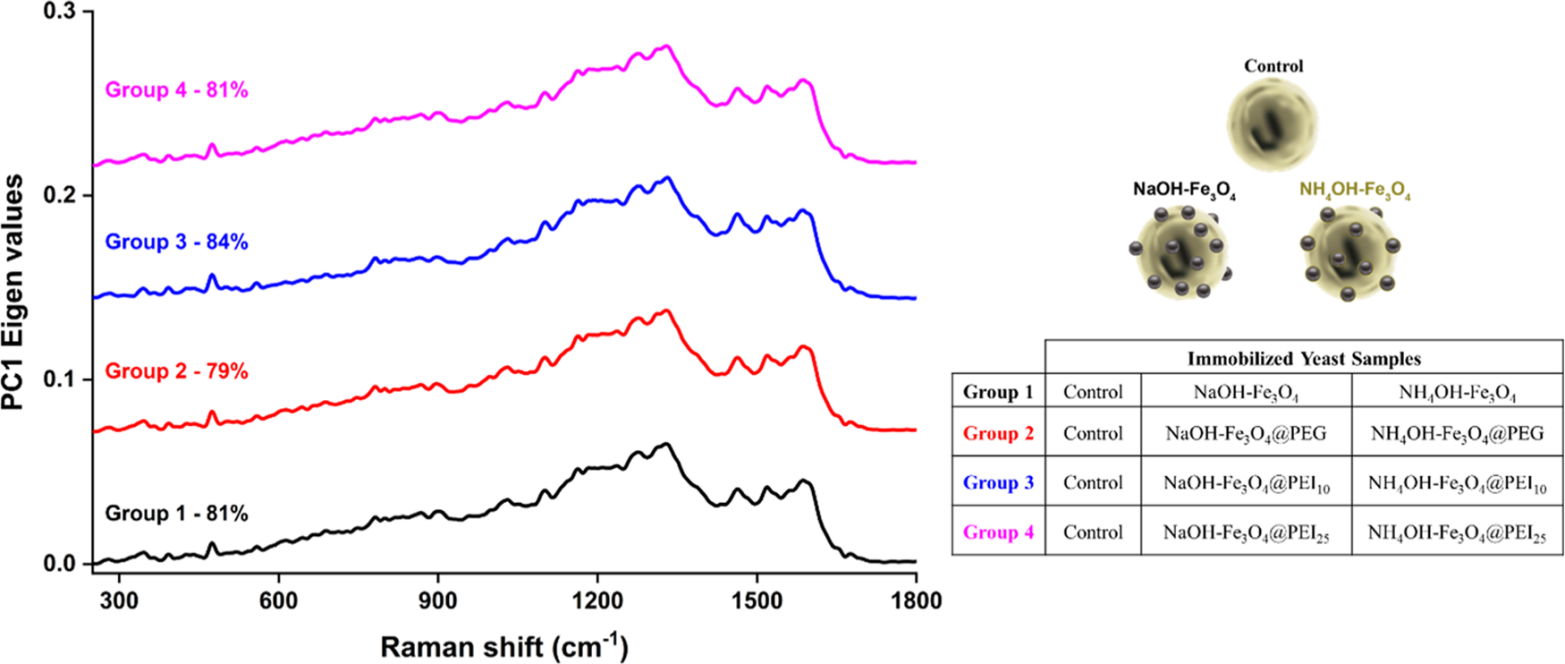
PC-1 loading graphs of magnetically immobilized cells. Groups are divided according to the base source. Order of graphs from top left (Group 1 to 4): control, NaOH–Fe_3_O_4_, NH_4_OH–Fe_3_O_4_; control, NaOH–Fe_3_O_4_@PEG, NH_4_OH–Fe_3_O_4_@PEG; control, NaOH–Fe_3_O_4_@PEI_10_, NH_4_OH–Fe_3_O_4_@PEI_10_; and control, NaOH–Fe_3_O_4_@PEI_25_, NH_4_OH–Fe_3_O_4_@PEI_25_ MNPs.

First, unsupervised ML analyses were utilized to determine similarities and differences between *K. phaffii* cells were immobilized with different MNPs. These analyses are employed to identify correlations, similarities, or disparities among data sets.^21,47,48^ For the case of PCA, first, we selected four groups, which were summarized in the insets of Figure 5, to determine their potential biochemical differentiation. As a result of the PCA, we obtained two-dimensional PCA plots for each group (Figure S5a). Here, the PC score values indicate the degree of discrimination between yeast cells within their groups. While the PC-1 number represents the highest discrimination level, the PC-2 value is relatively lower. The total score values (PC-1 + PC-2) obtained for Groups 1, −2, −3, and −4 were found to be 89, 88, 91, and 89%, respectively, indicating a high level of discrimination.

In the next step, HCA (Figure S5b) was performed to validate PCA by using the Ward algorithm. The distances between the specimens were visualized by a dendrogram chart resembling that of the rodent. While separate samples are located at opposite extremes of the distance, identical examples are intertwined. The resultant dendrogram (Figure S5b) graph clearly shows that most samples form separate links, and each sample is significantly separated from the control group. However, some samples (see NaOH–Fe_3_O_4_, NH_4_OH–Fe_3_O_4_) are intermixed, which is reasonably expected. Herein, note that our primary focus is to determine the change in the biomolecular nature of yeast cells after immobilization of MNPs with different surface molecules. Also, while the spectral data of the control group showed a more significant distribution, MNP-immobilized groups were concentrated in a significantly smaller area. This issue is mainly attributed to a certain differentiation between control and MNP-immobilized groups and the similar nature of MNP-immobilized groups in the biochemical signature.

In light of PC scores and HCA results, we detected that the Fe_3_O_4_@PEI_10_ MNP system showed the highest interaction between *K. phaffii* and MNPs and the resultant biomolecular change. In addition, in Figure 5, PCA loading plots were used to evaluate the spectral differences within each group. The average spectrum was similar to the load graphs obtained in this context (see and compare Figures 4 and 5). An examination of these spectra through qualitative and quantitative analyses unveiled both their similarities and their variances. The *x*-axis in these graphs shows the wavenumber, while the *y*-axis represents the eigenvalue (Figure 5). The high eigenvalue indicates the band where the discrimination is highly pronounced. The positive and negative scores in the loading plots present the extent of the contribution to variation, which increases or decreases, respectively.^21,47,55^ To further enhance the understanding of the contribution of PCA loading tables, the positions of the bands were identified, variance values were calculated for each PC-1 score, and the biomolecules corresponding to the Raman bands were assigned and are summarized in Table 1. In this table, we mainly focused on the Raman bands of each group with variance values higher than 80%, which indicates relatively more satisfying discrimination. From this data, for each group, we noticed various differentiations in the Raman bands associated with the biomolecular change in both yeast cell walls and cell membranes due to the interaction between cells and MNPs. First, the protein-originated Raman bands were observed to have the most remarkable differentiation and high variance. For all groups, we detected various biochemical changes in the nature of proteins for phenylalanine in proteins at 1049 cm^–1^, aromatic amino acids in proteins at 1183 cm^–1^, mannoprotein at 1313 cm^–1^, and C–H deformation vibrations from proteins at 1329 cm^–1^. Also, many bands relevant to Amide III were noticed at 1101 cm^–1^ and in the range of 1195–1275 cm^–1^. While the yeast cell wall has a high proportion of mannoproteins, which are proteoglycans with mainly protein and d-mannose content, the cell membrane has various membrane transporter proteins that regulate the transport activity^56,57^ (see Figure 4). Also, the differentiations in amide III bands can be attributed to the change in the α-helix and β-sheet structure motif of proteins.^58^ Considering their high protein content, it seems that the interaction between yeast cells and immobilized MNPs led to a dramatic change in the nature of proteins on the cell walls and membranes. In addition to proteins, we observed some differentiation associated with the change in *N*-acetylglucosamine characteristics. Two distinctive peaks were detected at 1162 and 1329 cm^–1^ with significantly high variances. *N*-acetylglucosamine is the main polysaccharide component of mannoproteins and chitin, which construct the main backbone of the yeast cell wall^59^ (see Figure 4). A significant number of peaks overlap in the spectral range of 1190–1385 cm^–1^, which are associated with nucleic acids, proteins, and lipids.^60,61^ Therefore, the peak at 1329 cm^–1^ may be attributed to lipids, specifically, C–H vibrations in cellular membrane lipids. This implies that lipids may also be involved in the interaction between MNPs and the cell surface. The 1162 cm^–1^ band was not observed in NaOH–Fe_3_O_4_, NaOH–Fe_3_O_4_@PEG, and NaOH–Fe_3_O_4_@PEI_10_ MNPs. This finding provides evidence of the hypothesis that the base source and polymer added to MNPs during production significantly impact the interaction between the yeast and MNPs. Like proteins, the immobilization of MNPs onto yeast cells dramatically manipulated the structure of mannoproteins and chitin via their polysaccharide content. Considering these analyses, we can conclude that unquestionable differentiation in the biomolecular signature of both cell wall and membrane led to undoubted manipulation in the permeability of yeast cell wall and membrane and resultant enhancement in the secretion of recombinant azurin protein from *K. phaffii* cells. Similarly, in a study, three different *Aspergillus* species captured with Fe_3_O_4_@PEI.NH_2_ MNPs exhibited significant Raman peak increases at 1218, 1326, 1457, and 1628 cm^–1^.^52^ The interaction of MNPs with yeast surface proteins may also be valid for different yeast species. Indeed, these bands are typical for *Candida* species, as well.^26^ However, the deeper mechanism behind these phenomena (MNP–yeast interaction) requires extensive experimental and theoretical studies, which will be the topic of future works.

**Table 1:** PC-1 Loading Peak Assignment of the Most Significant Peaks in the SERS Spectra of MNP-Immobilized *K. phaffii*

	groups, wavenumber (cm^–1^), variance (%)				
peak assignment (PC-1)	Group 1 control & NaOH–Fe_3_O_4_ & NH_4_OH–Fe_3_O_4_	Group 2 control & NaOH–Fe_3_O_4_@PEG & NH_4_OH–Fe_3_O_4_@PEG	Group 3 control & NaOH–Fe_3_O_4_@PEI_10_ & NH_4_OH–Fe_3_O_4_@PEI_10_	Group 4 control & NaOH–Fe_3_O_4_@PEI_25_ & NH_4_OH–Fe_3_O_4_@PEI_25_	refs
tyrosine, proline	866 (69%)				(28,51)
skeletal C–C, α-helix	900 (63%)				(28)
ν(C–C), ν(C–O) ν(C–N) of proteins	960 (72%)				(28,63)
*N*-acetylglucosamine ν(CO) + δ(CH)	998 (74%)	997 (76%)		997 (77%)	(28)
δ(CH), Phe	1030 (84%)	1030 (83%)	1030 (81%)	1030 (83%)	(64)
phenylalanine in proteins/C–O, C–N	1049 (84%)	1049 (84%)	1053 (81%)	1049 (83%)	(64)
amide III > PO2– str (sym)	1101 (85%)	1101 (86%)	1101 (87%)	1101 (84%)	(28)
					(65)
C–C stretch and C–O–C glycosidic link and symmetric ring breathing modes			1144 (92%)		(50)
C–H and −OH bending in mannose and *N*-acetylglucosamine	1163 (92%)	1163 (92%)	1163 (93%)	1163 (92%)	(26)
δ(Cs–H), C–O ring, aromatic amino acids in proteins	1183 (93%)	1183 (93%)	1183 (93%)	1183 (93%)	(66)
amide III, ν_as_(PO_2_^–^) str.	1195 (94%)	1195 (95%)	1193 (95%)	1190 (94%)	(28)
amide III, ν_as_(PO_2_^–^) str.	1209 (93%)	1210 (93%)	1206 (94%)	1212 (94%)	(28)
amide III	1236 (96%)	1236 (96%)	1235 (96%)	1236 (96%)	(28)
amide III	1275 (96%)	1275 (96%)	1277 (96%)	1275 (96%)	(66)
τ(CH_2_), w(CH_2_), lipids, proteins; ν(ring), Trp, tyr, Phe, proteins; mannoprotein		1313 (96%)	1314 (96%)	1313 (96%)	(63)
C–H deformation vibrations from proteins	1329 (95%)	1329 (93%)	1331 (94%)	1329 (94%)	(67)
β-1,4*N*-acetylglucosamine from mannose	1434 (87%)	1434 (85%)	1435 (92%)	1434 (87%)	(26)
nucleic acid modes	1463 (80%)	1463 (79%)	1463 (90%)	1463 (81%)	(28)
C–H_2_ def					(65)
lipids					(36)
ν_as_(COO^–^), proteins	1519 (61%)	1519 (61%)	1519 (64%)	1519 (63%)	(63)
ν_as_(COO^–^), proteins			1533 (79%)		(63)
C=C–C=C symmetric stretch of lipid	1565 (77%)	1565 (77%)	1561 (79%)	1565 (79%)	(68)
C=C bending mode of phe nucleic acids (DNA/RNA)	1584 (82%)	1584 (83%)	1585 (85%)	1587 (83%)	(28)
					(69)

**Figure 6:**
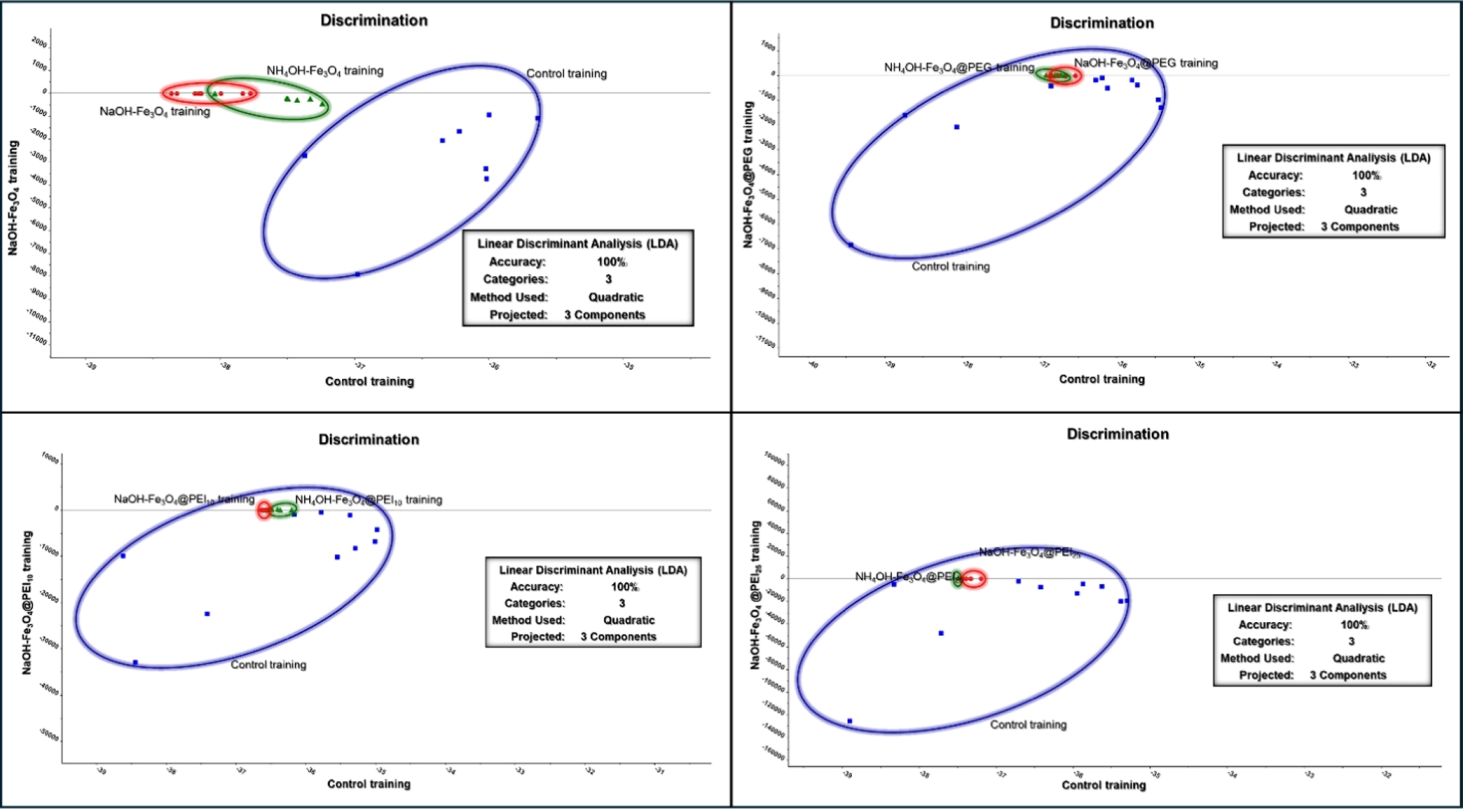
LDA discrimination of magnetically immobilized *K. phaffii*. Groups are divided according to the base source. Order of graphs from top left: control, NaOH–Fe_3_O_4_, NH_4_OH–Fe_3_O_4_; control, NaOH–Fe_3_O_4_@PEG, NH_4_OH–Fe_3_O_4_@PEG; control, NaOH–Fe_3_O_4_@PEI_10_, NH_4_OH–Fe_3_O_4_@PEI_10_; control, NaOH–Fe_3_O_4_@PEI_25_, NH_4_OH–Fe_3_O_4_@PEI_25_ MNPs.

After achieving successful outcomes in PCA-HCA, both linear and nonlinear supervised ML methods were employed to ascertain and validate the differentiation between the yeast cell groups. Within this context, each sample group has been provided with training sets and models formed from these sets. The test sets were randomly selected from the samples, and the analysis results were presented by using training models. LDA uses all of the spectral data and a single PCA model among these supervised ML analyses. In contrast, SVM analysis uses the bands with the highest variances and a PCA model for each band. Therefore, LDA can generate a more band-specific discrimination analysis than SVM. The LDA employs a single PCA model to concentrate on the distinction between different classes and optimize classification.^47,62^ The results of LDA were summarized in Figure 6 for each yeast cell group. A classification with 100% accuracy was obtained for each group, indicating the categorization of all test groups into their corresponding classes. This level of accuracy is also observed in the predicted and observed matrix table of the training data sets (see Tables S1–S4). Randomly selected test samples were classified into their classes (Tables S5–S8), which supported the accuracy of the 100% classification.

Finally, SVM analysis, which performs the classification regarding two spectral ranges in the *x*–*y* plane, was utilized. In this context, the PC loading graph showing the spectral range with the highest discrimination was selected as a reference. Figure S6 shows the SVM classification plots based on the spectral ranges 1329–31 and 1584–85–87 cm^–1^ for each sample group. The classification via SVM created over 90% validation and 100% accuracy. The accuracy is further confirmed by the anticipated and observed matrix tables for the training data sets (see Tables S9 and S12). Moreover, the test samples were classified into their corresponding classes, which were summarized in Tables S13–S16.

These supervised ML analyses showed that the SERS spectral data collected from MNP-immobilized yeast cells exhibited a 100% level of accuracy, sensitivity, and specificity, validating the reliability of the proposed approach and the high efficiency of GNAs as SERS platform.

## Conclusions

The bare, PEI-, and PEG-modified MNPs were synthesized in a one-step reaction with NH_4_OH or NaOH as a base source and characterized in depth. One-pot coprecipitation reaction is a simple method of creating surface-modified MNPs. All MNPs exhibited a magnetite-like crystal structure and excellent dispersion in aqueous solutions. The ζ-potential values of all MNPs created with NH_4_OH are more than +25 mV. However, only positive ζ-potential was found when 25 kDa PEI was employed in the NaOH synthesis. The most unstable MNPs with the highest aggregation tendency were produced using PEI in the presence of NaOH. It was shown that the size of MNPs may be decreased by increasing the length of the polymer or adding polymer to the reaction.

The molecular interaction of *K. phaffii* cells with the one-step produced and functionalized MNPs was investigated by recombinant protein secretion and ML-assisted SERS techniques. We effectively decorated the surface of *K. phaffii* cells with surface-modified MNPs and efficiently collected these magnetically immobilized cells using a simple magnet (>92% immobilization efficiency). The size, surface structure, and morphology of MNPs affect the adhesion of *K. phaffii* and protein secretion. Immobilized *K. phaffii* with MNPs synthesized using NH_4_OH showed a more significant Fe accumulation on the cell surface. The presence of PEG or PEI did not significantly alter the quantity of Fe on the surface of *K. phaffii* cells in terms of their immobilization with MNPs.

The findings suggest that using MNPs for cell immobilization significantly enhances the extracellular secretion of azurin. The highest protein secretion was observed in NH_4_OH–Fe_3_O_4_@PEI_10_ MNP-immobilized *K. phaffii* cells compared to nonimmobilized cells. As demonstrated by this study, the size and surface properties of MNPs mainly influence protein secretion, but not immobilization.

Our 3D GNAs SERS substrate exhibited excellent SERS signals from magnetically immobilized *K. phaffii* cells. Remarkable changes were seen in the SERS peaks of the yeast samples with and without magnetic immobilization. PCA loading plots were used to evaluate the spectral differences within each group. The best separation in ML-assisted SERS was noted in cells immobilized with NH_4_OH–Fe_3_O_4_@PEI_10_ MNPs, as seen in protein secretion performance. Protein-derived SERS peaks exhibited a significant distinction and crucial variance. The interaction between yeast cells and MNPs resulted in significant alteration of the proteins on the cell walls and membranes. Our investigation revealed significant changes in the biomolecular composition of the cell wall and cell membrane, resulting in a considerable enhancement in the secretion of recombinant protein from *K. phaffii*. ML algorithms, with their ability to quantify these changes, played a crucial role in offering insights into the mechanism of enhanced protein secretion. However, the mechanism underlying these events necessitates comprehensive experimental and theoretical investigations, which will be the focus of future research.

## Supporting Information Available

Dynamic light scattering (DLS) measurement used to determine the size distribution of the MNPs (PDI) in the solution and the hydrodynamic radius (*R*_h_) (Figure S1); magnetic attraction of MNPs in solution toward a magnet (Figure S2); cytotoxic effect of surface-modified MNPs on *K. phaffii* (Figure S3); characterization of SERS active surface (Figure S4), unsupervised ML analyses of SERS spectra (Figure S5); SVM classification plot for magnetically immobilized *K. phaffii* (Figure S6); prediction matrix of the training set performed by PCA-LDA for immobilized *K. phaffii* by Fe_3_O_4_ MNPs (Table S1); prediction matrix of the training set performed by PCA-LDA for immobilized *K. phaffii* by Fe_3_O_4_@PEG MNPs (Table S2); prediction matrix of the training set performed by PCA-LDA for immobilized *K. phaffii* by Fe_3_O_4_@PEI_10_ MNPs (Table S3); prediction matrix of the training set performed by PCA-LDA for immobilized *K. phaffii* by Fe_3_O_4_@PEI_25_ MNPs (Table S4); classification table of the validation test samples performed by PCA-LDA for Fe_3_O_4_ MNPs (Table S5); classification table of the validation test samples performed by PCA-LDA for Fe_3_O_4_@PEG MNPs (Table S6); classification table of the validation test samples performed by PCA-LDA for Fe_3_O_4_@PEI_10_ MNPs (Table S7); classification table of the validation test samples performed by PCA-LDA for Fe_3_O_4_@PEI_25_ MNPs (Table S8); prediction matrix of the training set realized by PCA-SVM for Fe_3_O_4_ MNPs (Table S9); prediction matrix of the training set realized by PCA-SVM for Fe_3_O_4_@PEG MNPs (Table S10); prediction matrix of the training set realized by PCA-SVM for Fe_3_O_4_@PEI_10_ MNPs (Table S11); prediction matrix of the training set realized by PCA-SVM for Fe_3_O_4_@PEI_25_ MNPs (Table 12); classification table of the validation test samples performed by PCA-SVM for Fe_3_O_4_ MNPs (Table S13); classification table of the validation test samples performed by PCA-SVM for Fe_3_O_4_@PEG MNPs (Table S14); classification table of the validation test samples performed by PCA-SVM for Fe_3_O_4_@PEI_10_ MNPs (Table S15); and classification table of the validation test samples performed by PCA-SVM for Fe_3_O_4_@PEI_25_ MNPs (Table S16) (PDF)
