# Supplementary material for: Machine Learning-Assisted SERS Reveals the Biochemical Signature of Enhanced Protein Secretion from Surface-Modified Magnetic Nanoparticles
Source: ACS Appl Mater Interfaces. 2024 Dec 11;16(51):70392–406. doi: 10.1021/acsami.4c18591 (PMC11672479; doi:10.1021/acsami.4c18591)
Supplement: Supplementary file 1 — am4c18591_si_001.pdf [file am4c18591_si_001.pdf]

Supporting Information for

Machine Learning-Assisted SERS Reveals the Biochemical Signature of Enhanced Protein Secretion from Surface-Modified Magnetic Nanoparticles

Ibrahim Dagci[1,#], *Kubra Solak*[2,3,4,#], *Nazli Oncer*[3,#], *Seyda Yildiz Arslan*[1,#], *Yagmur Unver*[2,4,*], *Mehmet Yilmaz*[2,3,5,*] *and Ahmet Mavi*[2,3,6,*]

[1]: Department of Molecular Biology and Genetics, Institute of Science and Technology, Atatürk University, 25240 Erzurum, Türkiye.

[2]: East Anatolia High Technology Application and Research Center (DAYTAM), Atatürk University, 25240 Erzurum, Türkiye.

[3]: Department of Nanoscience and Nanoengineering, Institute of Science and Technology, Atatürk University, 25240 Erzurum, Türkiye.

[4]: Department of Molecular Biology and Genetics, Faculty of Science, Atatürk University, 25240 Erzurum, Türkiye.

[5]: Department of Chemical Engineering, Atatürk University, 25240 Erzurum, Türkiye.

[6]: Department of Mathematics and Science Education, Education Faculty of Kazim Karabekir, Atatürk University, 25240 Erzurum, Türkiye.

[*]: Corresponding authors: yunver@atauni.edu.tr, mehmetylmz@atauni.edu.tr, amavi@atauni.edu.tr

[#]: I.D., K.S., N.O. and S.Y.A contributed equally to this work.

The file includes:

Figure S1. Dynamic Light Scattering (DLS) measurement is used to determine the size distribution of the MNPs (PDI) in the solution and the hydrodynamic radius (Rh).
Figure S2. Magnetic attraction of MNPs in solution toward a magnet.
Figure S3. The cytotoxic effect of surface-modified MNPs on *K. phaffii*.
Figure S4. Characterization of SERS active surface.
Figure S5. Unsupervised ML analyses of SERS spectra.
Figure S6. SVM classification plot for magnetically immobilized *K. phaffii*.
Table S1. Prediction matrix of the training set performed by PCA-LDA for immobilized *K. phaffii* by Fe_3O_4 MNPs.
Table S2. Prediction matrix of the training set performed by PCA-LDA for immobilized *K. phaffii* by Fe_3O_4@PEG MNPs.
Table S3. Prediction matrix of the training set performed by PCA-LDA for immobilized *K. phaffii* by Fe_3O_4@PEI_{10} MNPs.
Table S4. Prediction matrix of the training set performed by PCA-LDA for immobilized *K. phaffii* by Fe_3O_4@PEI_{25} MNPs.
Table S5. Classification table of the validation test samples performed by PCA-LDA for Fe_3O_4 MNPs.
Table S6. Classification table of the validation test samples performed by PCA-LDA for Fe_3O_4@PEG MNPs.
Table S7. Classification table of the validation test samples performed by PCA-LDA for Fe_3O_4@PEI_{10} MNPs.
Table S8. Classification table of the validation test samples performed by PCA-LDA for Fe_3O_4@PEI_{25} MNPs.
Table S9. Prediction matrix of the training set realized by PCA-SVM for Fe_3O_4 MNPs.
Table S10. Prediction matrix of the training set realized by PCA-SVM for Fe_3O_4@PEG MNPs.
Table S11. Prediction matrix of the training set realized by PCA-SVM for Fe_3O_4@PEI_{10} MNPs.
Table S12. Prediction matrix of the training set realized by PCA-SVM for Fe_3O_4@PEI_{25} MNPs.
Table S13. Classification table of the validation test samples performed by PCA-SVM for Fe_3O_4 MNPs.
Table S14. Classification table of the validation test samples performed by PCA-SVM for Fe_3O_4@PEG MNPs.
Table S15. Classification table of the validation test samples performed by PCA-SVM for Fe_3O_4@PEI_{10} MNPs.
Table S16. Classification table of the validation test samples performed by PCA-SVM for Fe_3O_4@PEI_{25} MNPs.

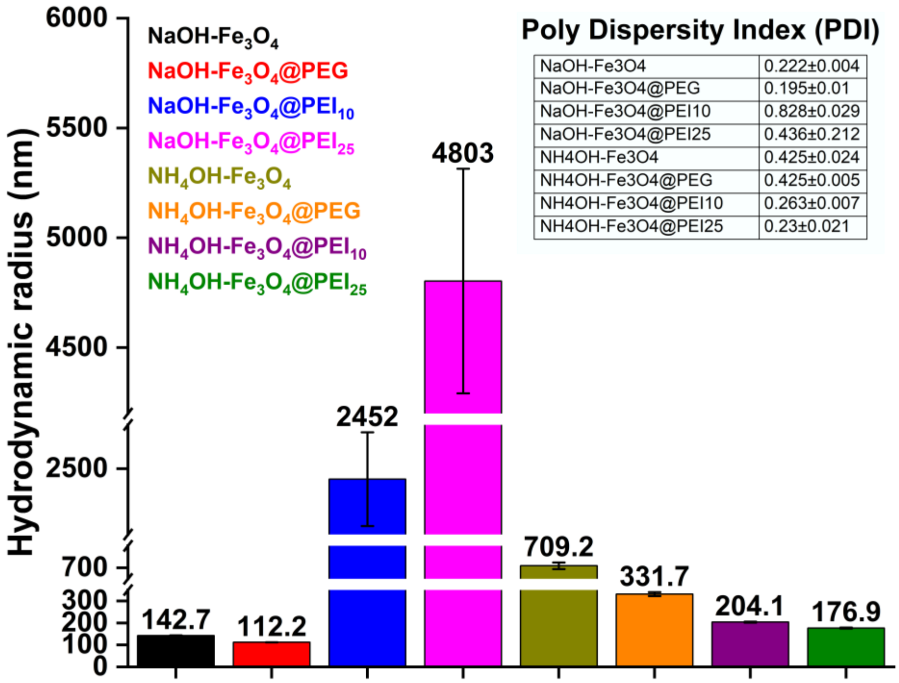

Figure S1. Dynamic Light Scattering (DLS) measurement is used to determine the size distribution of the MNPs (PDI) in the solution and the hydrodynamic radius (Rh).

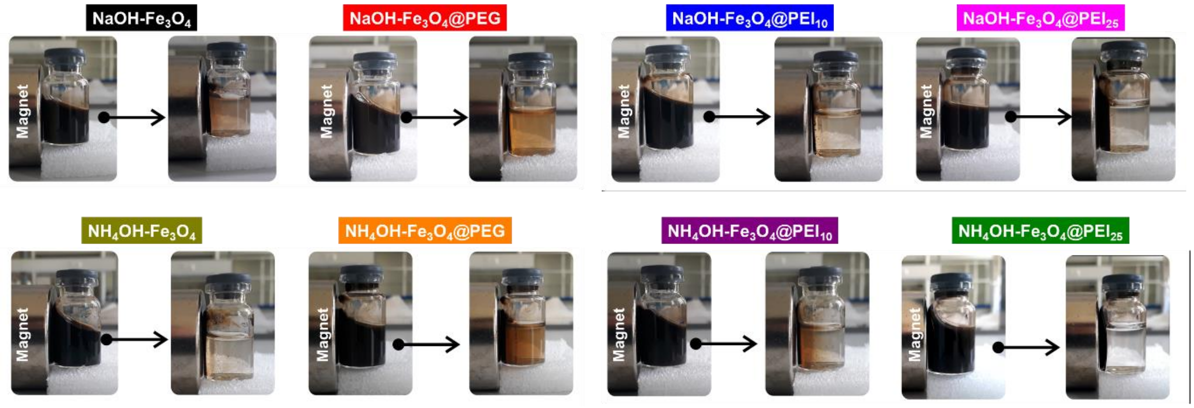

Figure S2. Magnetic attraction of MNPs in solution toward a magnet.

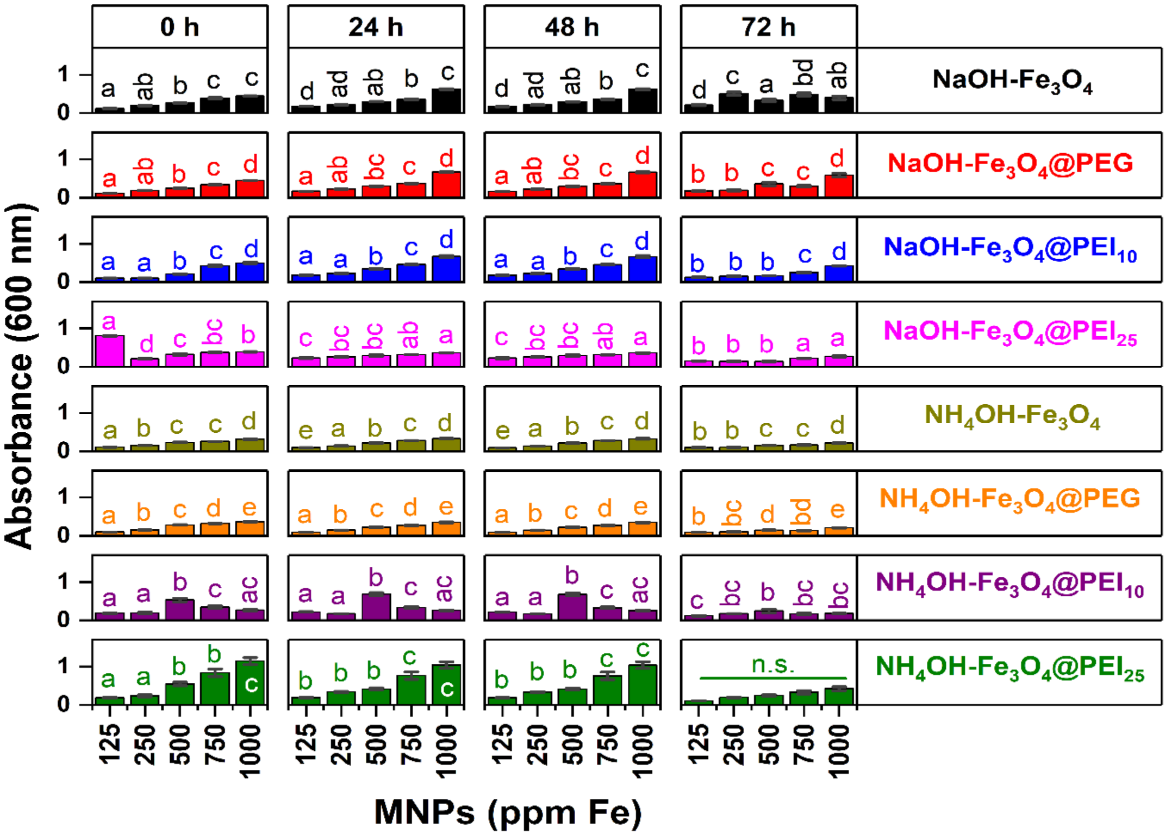

Figure S3. The cytotoxic effect of surface-modified MNPs on *K. phaffii*. Optic density (OD_{600}) measurement of magnetically immobilized *K. phaffii* at different concentrations and incubation times.

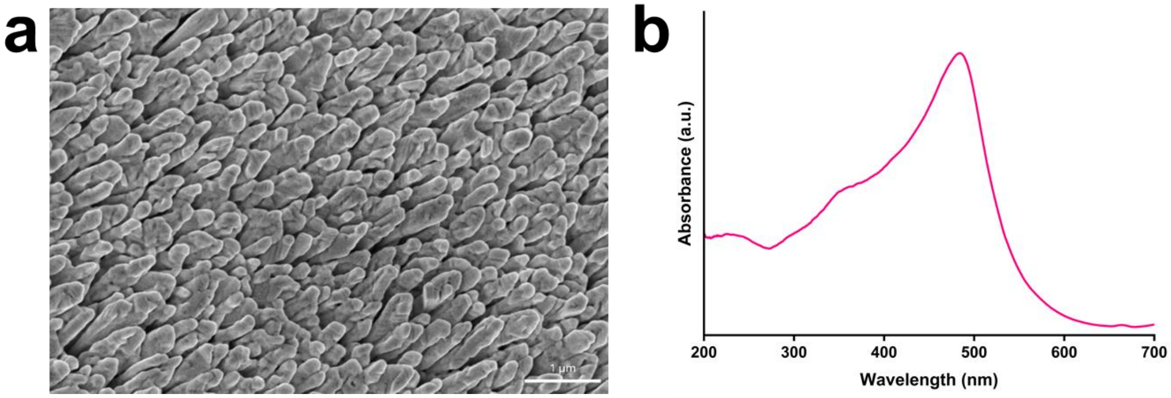

Figure S4. Characterization of SERS active surface. a. SEM image and b. UV–vis absorption spectra of GNAs. The scale bar is 1 µm.

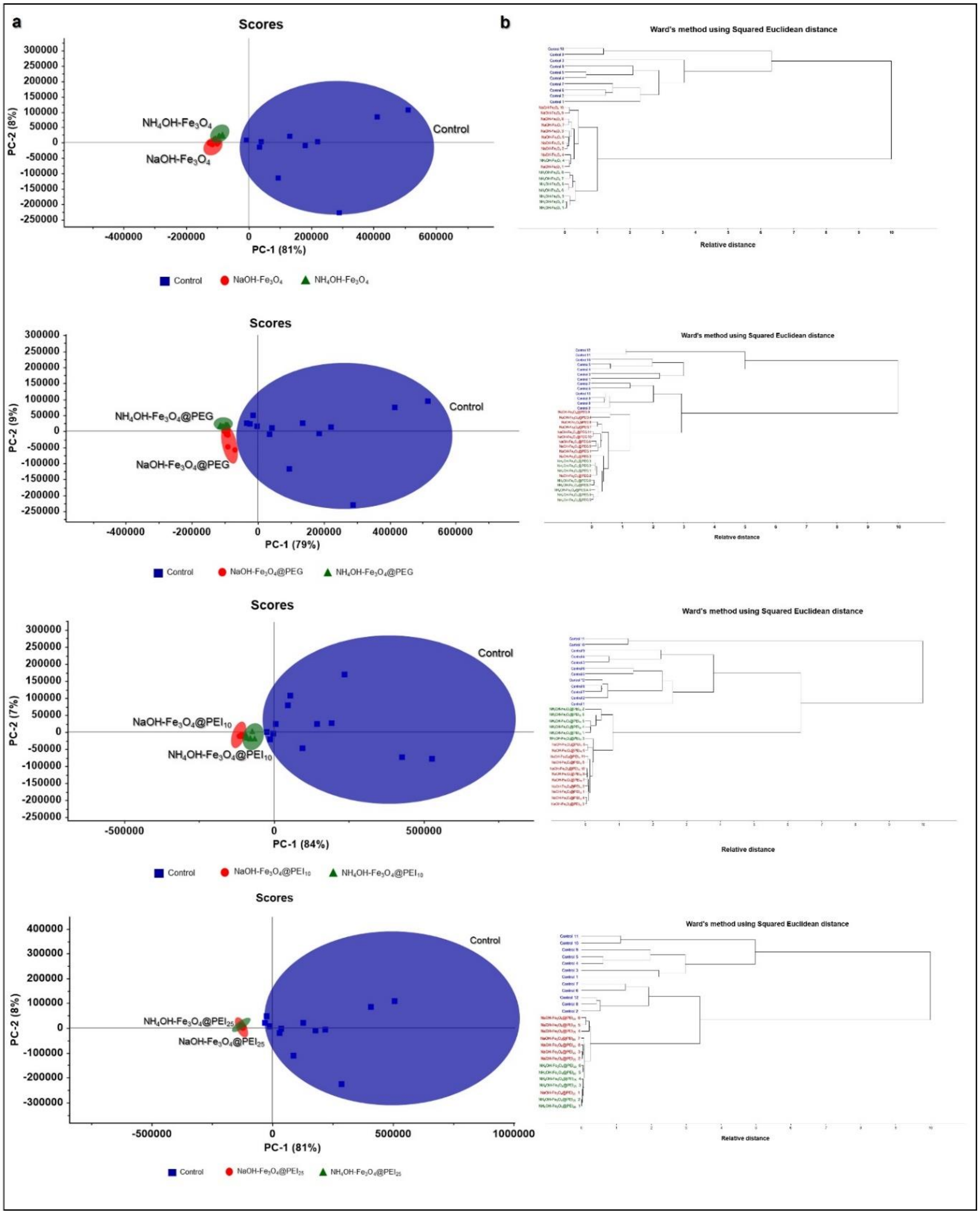

Figure S5. Unsupervised ML analyses of SERS spectra. a. PCA score plots and b. HCA dendrograms belong to magnetically immobilized *K. phaffii*. Groups are divided according to the base source. Order of graphs from top left: control, NaOH-Fe_3O_4, NH_4OH-Fe_3O_4; control, NaOH-Fe_3O_4@PEG, NH_4OH-Fe_3O_4@PEG; control, NaOH-Fe_3O_4@PEI_{10}, NH_4OH-Fe_3O_4@PEI_{10}; and control, NaOH-Fe_3O_4@PEI_{25}, NH_4OH-Fe_3O_4@PEI_{25} MNPs.

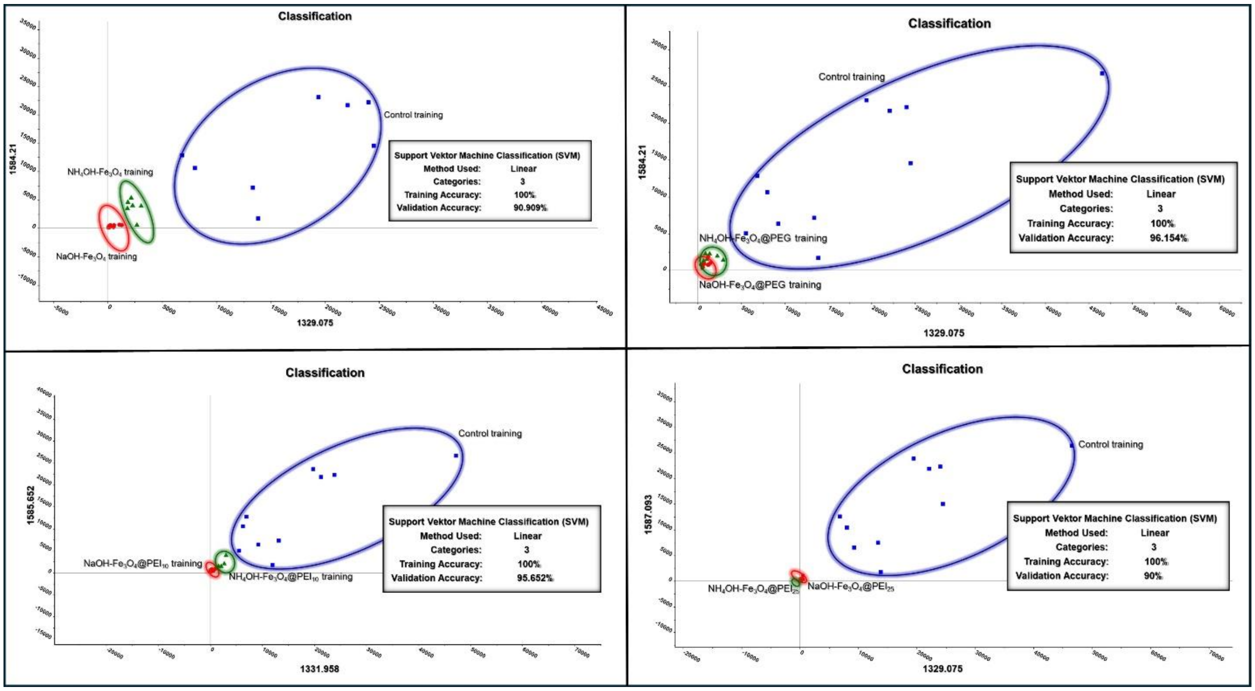

Figure S6. SVM classification plot for magnetically immobilized *K. phaffii*. Groups are divided according to the base source. Order of graphs from top left: control, NaOH-Fe_3O_4, NH_4OH-Fe_3O_4; control, NaOH-Fe_3O_4@PEG, NH_4OH- Fe_3O_4@PEG; control, NaOH-Fe_3O_4@PEI_{10}, NH_4OH-Fe_3O_4@PEI_{10}; and control, NaOH-Fe_3O_4@PEI_{25}, NH_4OH- Fe_3O_4@PEI_{25} MNPs.

Table S1. Prediction matrix of the training set performed by PCA-LDA for immobilized *K. phaffii* by Fe_3O_4 MNPs. This matrix contains the predicted numbers for each combination of observed groups (rows) and predicted groups (columns).

**Prediction**					**Predicted**
	**Name of Samples**	**Control**	**NaOH-Fe_3O_4**	**NH_4OH-Fe_3O_4**	
**Control**	Control 1	-36.22	-1679.2	-13557.3	**Control**
	Control 2	-36.00	-964.6	-408.9	
	Control 3	-37.37	-2738.2	-44250.3	
	Control 4	-36.02	-3309.5	-2237.5	
	Control 5	-36.02	-3730.2	-706.4	
	Control 6	-35.64	-1112.8	-8176.9	
	Control 7	-36.35	-2070.8	-12124.5	
	Control 8	-36.98	-7923.9	-12526.2	
**NaOH-Fe_3O_4**	NaOH-Fe_3O_4 3	-37.99	-28.2	-185.1	**NaOH-Fe_3O_4**
	NaOH-Fe_3O_4 4	-38.14	-26.2	-69.1	
	NaOH-Fe_3O_4 5	-38.16	-25.5	-89.3	
	NaOH-Fe_3O_4 6	-38.32	-26.7	-46.6	
	NaOH-Fe_3O_4 7	-38.19	-27.5	-119.7	
	NaOH-Fe_3O_4 8	-38.36	-26.6	-51.1	
	NaOH-Fe_3O_4 9	-37.77	-27.0	-189.2	
	NaOH-Fe_3O_4 10	-37.83	-26.4	-183.9	
**NH_4OH-Fe_3O_4**	NH_4OH-Fe_3O_4 1	-37.49	-290.4	-26.6	**NH_4OH-Fe_3O_4**
	NH_4OH-Fe_3O_4 2	-37.43	-345.0	-26.5	
	NH_4OH-Fe_3O_4 3	-37.24	-514.1	-27.1	
	NH_4OH-Fe_3O_4 4	-38.03	-63.8	-27.9	
	NH_4OH-Fe_3O_4 5	-37.33	-297.4	-28.2	
	NH_4OH-Fe_3O_4 6	-37.50	-257.1	-28.0	

Table S2. Prediction matrix of the training set performed by PCA-LDA for immobilized *K. phaffii* by Fe_3O_4@PEG MNPs. This matrix contains the predicted numbers for each combination of observed groups (rows) and predicted groups (columns).

**Prediction**					**Predicted**
	**Name of Samples**	**Control**	**NaOH-Fe_3O_4@PEG**	**NH_4OH-Fe_3O_4@PEG**	
**Control**	Control 1	-36.85	-438.35	-24932.8	**Control**
	Control 2	-35.80	-201.41	-299.3	
	Control 3	-38.74	-1608.60	-63890.3	
	Control 4	-35.43	-1316.96	-5033.4	
	Control 5	-35.46	-980.21	-1627.3	
	Control 6	-35.73	-384.99	-2005.7	
	Control 7	-36.12	-512.08	-3410.5	
	Control 8	-36.19	-114.19	-105.8	
	Control 9	-36.27	-194.35	-159.4	
	Control 10	-38.07	-2089.84	-7313.8	
	Control 11	-39.43	-6849.92	-19102.9	
**NaOH-Fe_3O_4@PEG**	NaOH-Fe_3O_4@PEG 3	-36.75	-29.49	-74.5	**NaOH-Fe_3O_4@PEG**
	NaOH-Fe_3O_4@PEG 4	-36.82	-31.16	-2465.4	
	NaOH-Fe_3O_4@PEG 5	-36.73	-28.93	-59.6	
	NaOH-Fe_3O_4@PEG 6	-36.77	-29.39	-115.1	
	NaOH-Fe_3O_4@PEG 7	-36.53	-30.40	-395.6	
	NaOH-Fe_3O_4@PEG 8	-36.65	-28.93	-331.3	
	NaOH-Fe_3O_4@PEG 9	-36.67	-31.41	-3776.8	
	NaOH-Fe_3O_4@PEG 10	-36.80	-29.43	-400.8	
	NaOH-Fe_3O_4@PEG 11	-36.85	-29.56	-280.0	
**NH_4OH-Fe_3O_4@PEG**	NH_4OH-Fe_3O_4@PEG 1	-36.68	-31.92	-26.1	**NH_4OH-Fe_3O_4@PEG**
	NH_4OH-Fe_3O_4@PEG 2	-36.71	-30.63	-26.2	
	NH_4OH-Fe_3O_4@PEG 3	-36.77	-29.69	-25.0	
	NH_4OH-Fe_3O_4@PEG 4	-36.91	-30.57	-26.0	
	NH_4OH-Fe_3O_4@PEG 5	-36.84	-29.57	-25.3	
	NH_4OH-Fe_3O_4@PEG 6	-36.79	-29.31	-25.8	

Table S3. Prediction matrix of the training set performed by PCA-LDA for immobilized *K. phaffii* by Fe_3O_4@PEI_{10} MNPs. This matrix contains the predicted numbers for each combination of observed groups (rows) and predicted groups (columns).

Prediction					Predicted
	**Name of Samples**	**Control**	**NaOH-Fe_3O_4@PEI_{10}**	**NH_4OH-Fe_3O_4@PEI_{10}**	
**Control**	Control 1	-38.62	-9935.81	-7788.01	**Control**
	Control 2	-35.36	-1151.59	-263.74	
	Control 3	-35.00	-6873.99	-2102.12	
	Control 4	-34.98	-4290.68	-873.00	
	Control 5	-35.29	-8338.40	-3316.47	
	Control 6	-35.54	-10184.14	-3199.11	
	Control 7	-35.78	-471.74	-55.19	
	Control 8	-36.16	-1084.08	-191.00	
	Control 9	-37.41	-22521.86	-6786.86	
	Control 10	-38.44	-32998.68	-2387.49	
**NaOH-Fe_3O_4@PEI_{10}**	NaOH-Fe_3O_4@PEI_{10} 3	-36.60	-23.81	-33.04	**NaOH-Fe_3O_4@PEI_{10}**
	NaOH-Fe_3O_4@PEI_{10} 4	-36.59	-23.48	-32.50	
	NaOH-Fe_3O_4@PEI_{10} 5	-36.55	-25.05	-32.02	
	NaOH-Fe_3O_4@PEI_{10} 6	-36.57	-23.98	-32.71	
	NaOH-Fe_3O_4@PEI_{10} 7	-36.62	-24.03	-32.09	
	NaOH-Fe_3O_4@PEI_{10} 8	-36.64	-24.73	-32.81	
	NaOH-Fe_3O_4@PEI_{10} 9	-36.56	-25.11	-31.30	
	NaOH-Fe_3O_4@PEI_{10} 10	-36.59	-25.20	-32.08	
	NaOH-Fe_3O_4@PEI_{10} 11	-36.53	-26.36	-30.56	
**NH_4OH-Fe_3O_4@PEI_{10}**	NH_4OH-Fe_3O_4@PEI_{10} 1	-36.36	-140.13	-28.32	**NH_4OH-Fe_3O_4@PEI_{10}**
	NH_4OH-Fe_3O_4@PEI_{10}-2	-36.20	-108.50	-28.32	
	NH_4OH-Fe_3O_4@PEI_{10} 3	-36.40	-39.81	-28.32	
	NH_4OH-Fe_3O_4@PEI_{10} 4	-36.48	-77.50	-28.32	

Table S4. Prediction matrix of the training set performed by PCA-LDA for immobilized *K. phaffii* by Fe_3O_4@PEI_{25} MNPs. This matrix contains the predicted numbers for each combination of observed groups (rows) and predicted groups (columns).

**Prediction**					**Predicted**
	**Name of Samples**	**Control**	**NaOH-Fe_3O_4@PEI_{25}**	**NH_4OH-Fe_3O_4@PEI_{25}**	
**Control**	Control 1	-36.71	-2325.15	-2077211.38	**Control**
	Control 2	-35.87	-4922.08	-3905412.50	
	Control 3	-38.32	-5138.95	-35501528.00	
	Control 4	-35.30	-19711.60	-10967888.00	
	Control 5	-35.38	-20058.86	-6992150.00	
	Control 6	-35.62	-7216.77	-21861078.00	
	Control 7	-35.94	-13023.50	-32432000.00	
	Control 8	-36.42	-7567.59	-320857.66	
	Control 9	-37.72	-48243.11	-79611000.00	
	Control 10	-38.90	-125659.84	-4531910.00	
**NaOH-Fe_3O_4@PEI_{25}**	NaOH-Fe_3O_4@PEI_{25} 3	-37.43	-25.94	-1599.01	**NaOH-Fe_3O_4@PEI_{25}**
	NaOH-Fe_3O_4@PEI_{25} 4	-37.33	-26.48	-7402.09	
	NaOH-Fe_3O_4@PEI_{25} 5	-37.19	-26.32	-241431.45	
	NaOH-Fe_3O_4@PEI_{25} 6	-37.33	-24.98	-36865.64	
	NaOH-Fe_3O_4@PEI_{25} 7	-37.39	-25.11	-24436.08	
	NaOH-Fe_3O_4@PEI_{25} 8	-37.44	-25.53	-5967.77	
**NH_4OH-Fe_3O_4@PEI_{25}**	NH_4OH-Fe_3O_4@PEI_{25} 1	-37.48	-26.64	-17.67	**NH_4OH-Fe_3O_4@PEI_{25}**
	NH_4OH-Fe_3O_4@PEI_{25} 2	-37.48	-27.18	-17.68	
	NH_4OH-Fe_3O_4@PEI_{25} 3	-37.49	-32.37	-17.67	
	NH_4OH-Fe_3O_4@PEI_{25} 4	-37.50	-37.78	-17.67	

Table S5. Classification table of the validation test samples performed by PCA-LDA for Fe_3O_4 MNPs. This table shows the relationship between the observed and predicted classes in the validation test.

Class		Control	NaOH-Fe_3O_4	NH_4OH-Fe_3O_4	Class
	Number of Samples	1	2	3	
Control 9	1	-42.65	-11532.44	-6734.87	Control
Control 10	2	-39.69	-7941.90	-4698.93	Control
NaOH-Fe_3O_4 1	3	-37.94	-36.08	-97.38	NaOH-Fe_3O_4
NaOH-Fe_3O_4 2	4	-38.20	-26.29	-83.62	NaOH-Fe_3O_4
NH_4OH-Fe_3O_4 7	5	-37.74	-179.40	-27.17	NH_4OH-Fe_3O_4
NH_4OH-Fe_3O_4 8	6	-37.53	-251.60	-26.16	NH_4OH-Fe_3O_4

Table S6. Classification table of the validation test samples performed by PCA-LDA for Fe_3O_4@PEG MNPs. This table shows the relationship between the observed and predicted classes in the validation test.

Class		Control	NaOH-Fe_3O_4@PEG	NH_4OH-Fe_3O_4@PEG	Class
	Number of Samples	1	2	3	
Control 12	1	-37.85	-4734.07	-15035.37	Control
Control 13	2	-36.38	-141.57	-641.98	Control
NaOH-Fe_3O_4@PEG 1	3	-36.60	-29.97	-111.53	NaOH-Fe_3O_4@PEG
NaOH-Fe_3O_4@PEG 2	4	-36.79	-29.92	-65.48	NaOH-Fe_3O_4@PEG
NH_4OH-Fe_3O_4@PEG 7	5	-37.04	-32.57	-43.77	NH_4OH-Fe_3O_4@PEG
NH_4OH-Fe_3O_4@PEG 8	6	-37.02	-32.43	-41.55	NH_4OH-Fe_3O_4@PEG

Table S7. Classification table of the validation test samples performed by PCA-LDA for Fe_3O_4@PEI_{10} MNPs. This table shows the relationship between the observed and predicted classes in the validation test.

Class		Control	NaOH-Fe_3O_4@PEI_{10}	NH_4OH-Fe_3O_4@PEI_{10}	Class
	Number of Samples	1	2	3	
**Control 11**	1	-37.02	-22659.05	-1784.08	**Control**
**Control 12**	2	-35.88	-750.68	-62.00	**Control**
**NaOH-Fe_3O_4@PEI_{10} 1**	3	-36.62	-24.36	-32.04	**NaOH-Fe_3O_4@PEI_{10}**
**NaOH-Fe_3O_4@PEI_{10} 2**	4	-36.61	-28.74	-33.53	**NaOH-Fe_3O_4@PEI_{10}**
**NH_4OH-Fe_3O_4@PEI_{10} 5**	5	-36.35	-113.49	-31.45	**NH_4OH-Fe_3O_4@PEI_{10}**
**NH_4OH-Fe_3O_4@PEI_{10} 6**	6	-36.14	-206.01	-83.44	**NH_4OH-Fe_3O_4@PEI_{10}**

Table S8. Classification table of the validation test samples performed by PCA-LDA for Fe_3O_4@PEI_{25} MNPs. This table shows the relationship between the observed and predicted classes in the validation test.

Class		Control	NaOH-Fe_3O_4@PEI_{25}	NH_4OH-Fe_3O_4@PEI_{25}	Class
	Number of Samples	1	2	3	
**Control 11**	1	-37.46	-83330.84	-3258631.25	**Control**
**Control 12**	2	-36.57	-2721.98	-116095.23	**Control**
**NaOH-Fe_3O_4@PEI_{25} 1**	3	-37.50	-30.62	-72.02	**NaOH-Fe_3O_4@PEI_{25}**
**NaOH-Fe_3O_4@PEI_{25} 2**	4	-37.43	-25.29	-2050.57	**NaOH-Fe_3O_4@PEI_{25}**
**NH_4OH-Fe_3O_4@PEI_{25} 5**	5	-37.44	-30.99	-6696.72	**NH_4OH-Fe_3O_4@PEI_{25}**
**NH_4OH-Fe_3O_4@PEI_{25} 6**	6	-37.47	-26.06	-54.36	**NH_4OH-Fe_3O_4@PEI_{25}**

Table S9. Prediction matrix of the training set realized by PCA-SVM for Fe_3O_4 MNPs. This matrix contains the predicted numbers for each combination of observed groups (rows) and predicted groups (columns).

**Prediction**	**Name of Samples**	**Predicted**
**Control**	Control 1	**Control**
	Control 2	
	Control 3	
	Control 4	
	Control 5	
	Control 6	
	Control 7	
	Control 8	
**NaOH-Fe_3O_4**	NaOH-Fe_3O_4 3	**NaOH-Fe_3O_4**
	NaOH-Fe_3O_4 4	
	NaOH-Fe_3O_4 5	
	NaOH-Fe_3O_4 6	
	NaOH-Fe_3O_4 7	
	NaOH-Fe_3O_4 8	
	NaOH-Fe_3O_4 9	
	NaOH-Fe_3O_4 10	
**NH_4OH-Fe_3O_4**	NH_4OH-Fe_3O_4 1	**NH_4OH-Fe_3O_4**
	NH_4OH-Fe_3O_4 2	
	NH_4OH-Fe_3O_4 3	
	NH_4OH-$Fe_3O_4$4	
	NH_4OH-Fe_3O_4 5	
	NH_4OH-Fe_3O_4 6	

Table S10. Prediction matrix of the training set realized by PCA-SVM for Fe_3O_4@PEG MNPs. This matrix contains the predicted numbers for each combination of observed groups (rows) and predicted groups (columns).

Prediction	Name of Samples	Predicted
**Control**	Control 1	**Control**
	Control 2	
	Control 3	
	Control 4	
	Control 5	
	Control 6	
	Control 7	
	Control 8	
	Control 9	
	Control 10	
	Control 11	
**NaOH-Fe_3O_4@PEG**	NaOH-Fe_3O_4@PEG 3	**NaOH-Fe_3O_4@PEG**
	NaOH-Fe_3O_4@PEG 4	
	NaOH-Fe_3O_4@PEG 5	
	NaOH-Fe_3O_4@PEG 6	
	NaOH-Fe_3O_4@PEG 7	
	NaOH-Fe_3O_4@PEG 8	
	NaOH-Fe_3O_4@PEG 9	
	NaOH-Fe_3O_4@PEG 10	
	NaOH-Fe_3O_4@PEG 11	
**NH_4OH-Fe_3O_4@PEG**	NH_4OH-Fe_3O_4@PEG 1	**NH_4OH-Fe_3O_4@PEG**
	NH_4OH-Fe_3O_4@PEG 2	
	NH_4OH-Fe_3O_4@PEG 3	
	NH_4OH-Fe_3O_4@PEG 4	
	NH_4OH-Fe_3O_4@PEG 5	
	NH_4OH-Fe_3O_4@PEG 6	

Table S11. Prediction matrix of the training set realized by PCA-SVM for Fe_3O_4@PEI_{10} MNPs. This matrix contains the predicted numbers for each combination of observed groups (rows) and predicted groups (columns).

Prediction	Name of Samples	Predicted
**Control**	Control 1	**Control**
	Control 1	
	Control 2	
	Control 3	
	Control 4	
	Control 5	
	Control 6	
	Control 7	
	Control 8	
	Control 9	
	Control 10	
**NaOH-Fe_3O_4@PEI_{10}**	NaOH-Fe_3O_4@PEI_{10} 3	**NaOH-Fe_3O_4@PEI_{10}**
	NaOH-Fe_3O_4@PEI_{10} 4	
	NaOH-Fe_3O_4@PEI_{10} 5	
	NaOH-Fe_3O_4@PEI_{10} 6	
	NaOH-Fe_3O_4@PEI_{10} 7	
	NaOH-Fe_3O_4@PEI_{10} 8	
	NaOH-Fe_3O_4@PEI_{10} 9	
	NaOH-Fe_3O_4@PEI_{10} 10	
	NaOH-Fe_3O_4@PEI_{10} 11	
**NH_4OH-Fe_3O_4@PEI_{10}**	NH_4OH-Fe_3O_4@PEI_{10} 1	**NH_4OH-Fe_3O_4@PEI_{10}**
	NH_4OH-Fe_3O_4@PEI_{10} 2	
	NH_4OH-Fe_3O_4@PEI_{10} 3	
	NH_4OH-Fe_3O_4@PEI_{10} 4	

Table S12. Prediction matrix of the training set realized by PCA-SVM for Fe_3O_4@PEI_{25} MNPs. This matrix contains the predicted numbers for each combination of observed groups (rows) and predicted groups (columns).

Prediction	Name of Samples	Predicted
**Control**	Control 1	**Control**
	Control 2	
	Control 3	
	Control 4	
	Control 5	
	Control 6	
	Control 7	
	Control 8	
	Control 9	
	Control 10	
**NaOH-Fe_3O_4@PEI_{25}**	NaOH-Fe_3O_4@PEI_{25} 3	**NaOH-Fe_3O_4@PEI_{25}**
	NaOH-Fe_3O_4@PEI_{25} 4	
	NaOH-Fe_3O_4@PEI_{25} 5	
	NaOH-Fe_3O_4@PEI_{25} 6	
	NaOH-Fe_3O_4@PEI_{25} 7	
	NaOH-Fe_3O_4@PEI25 8	
**NH_4OH-Fe_3O_4@PEI_{25}**	NH_4OH-Fe_3O_4@PEI_{25} 1	**NH_4OH-Fe_3O_4@PEI_{25}**
	NH_4OH-Fe_3O_4@PEI_{25} 2	
	NH_4OH-Fe_3O_4@PEI_{25} 3	
	NH_4OH-Fe_3O_4@PEI_{25} 4	
	NH_4OH-Fe_3O_4@PEI_{25} 3	
	NH_4OH-Fe_3O_4@PEI_{25} 4	

Table S13. Classification table of the validation test samples performed by PCA-SVM for Fe_3O_4 MNPs. This table shows the relationship between the observed and predicted classes in the validation test.

Class	Number of Samples	Class
**Control 9**	1	**Control**
**Control 10**	2	**Control**
**NaOH-Fe_3O_4 1**	3	**NaOH-Fe_3O_4**
**NaOH-Fe_3O_4 2**	4	**NaOH-Fe_3O_4**

**NH_4OH-Fe_3O_4 7**	5	**NH_4OH-Fe_3O_4**
**NH_4OH-Fe_3O_4 8**	6	**NH_4OH-Fe_3O_4**

Table S14. Classification table of the validation test samples performed by PCA-SVM for Fe_3O_4@PEG MNPs. This table shows the relationship between the observed and predicted classes in the validation test.

Class	Number of Samples	Class
**Control 12**	1	**Control**
**Control 13**	2	**Control**
**NaOH-Fe_3O_4@PEG 1**	3	**NaOH-Fe_3O_4@PEG**
**NaOH-Fe_3O_4@PEG 2**	4	**NaOH-Fe_3O_4@PEG**
**NH_4OH-Fe_3O_4@PEG 7**	5	**NH_4OH-Fe_3O_4@PEG**
**NH_4OH-Fe_3O_4@PEG 8**	6	**NH_4OH-Fe_3O_4@PEG**

Table S15. Classification table of the validation test samples performed by PCA-SVM for Fe_3O_4@PEI_{10} MNPs. This table shows the relationship between the observed and predicted classes in the validation test.

Class	Number of Samples	Class
**Control 11**	1	**Control**
**Control 12**	2	**Control**
**NaOH-Fe_3O_4@PEI_{10} 1**	3	**NaOH-Fe_3O_4@PEI_{10}**
**NaOH-Fe_3O_4@PEI_{10} 2**	4	**NaOH-Fe_3O_4@PEI_{10}**
**NH_4OH-Fe_3O_4@PEI_{10} 5**	5	**NH_4OH-Fe_3O_4@PEI_{10}**
**NH_4OH-Fe_3O_4@ PEI_{10} 6**	6	**NH_4OH-Fe_3O_4@PEI_{10}**

Table S16. Classification table of the validation test samples performed by PCA-SVM for Fe_3O_4@PEI_{25} MNPs. This table shows the relationship between the observed and predicted classes in the validation test.

Class	Number of Samples	Class
**Control 11**	1	**Control**
**Control 12**	2	**Control**
**NaOH-Fe_3O_4@PEI_{25} 1**	3	**NaOH-Fe_3O_4@PEI_{25}**
**NaOH-Fe_3O_4@PEI_{25} 2**	4	**NaOH-Fe_3O_4@PEI_{25}**
**NH_4OH-Fe_3O_4@ PEI_{25} 5**	5	**NH_4OH-Fe_3O_4@PEI_{25}**
**NH_4OH-Fe_3O_4@ PEI_{25} 6**	6	**NH_4OH-Fe_3O_4@PEI_{25}**
